# Suitable indicators to determine tsunami impact on coastal areas in Northern Japan, Aomori Prefecture

**DOI:** 10.1007/s10661-022-09989-4

**Published:** 2022-04-20

**Authors:** Mike Frenken, Piero Bellanova, Yuichi Nishimura, Philipp Schulte, Frank Lehmkuhl, Klaus Reicherter, Jan Schwarzbauer

**Affiliations:** 1 Institute for Geology and Geochemistry of Petroleum and Coal, RWTH Aachen University, Lochnerstrasse 4-20, 52056 Aachen, Germany; 2 Neotectonics and Natural Hazards Group, RWTH Aachen University, Lochnerstrasse 4-20, 52056 Aachen, Germany; 3 Institute of Seismology and Volcanology, Hokkaido University, Kita-10, Nishi-8, Kita-ku, Sapporo, 060-0810 Japan; 4 Department of Geography, RWTH Aachen University, Wüllnerstrasse 5B, 52056 Aachen, Germany

**Keywords:** 2011 Tohoku-oki tsunami, Organic-geochemical signature, Tsunami indicators, Anthropogenic marker, Natural compounds, Tsunami geochemistry

## Abstract

The 2011 Tohoku-oki tsunami released and mobilized many anthropogenic and natural organic compounds and, hereby, left a clear signature in its sedimentary remnants. In this study, a wide variety of organic marker substances were analyzed in 15 sediment profiles from the Aomori coast (Northern Japan). Total organic carbon (TOC) and fine grain fraction normalization have been tested with the wide dataset, and the already more frequently used TOC normalization was proven to be the more suitable one. Concentration profiles and specific ratios have been interpreted using two different approaches. Differentiation of marine and terrestrial matter characterized mixing processes due to the tsunami impact. Linking constituents to anthropogenic emission sources pointed not only to pollution revealed by the tsunami damages but also to dispersion processes, in particular erosion, transport, mixing and redeposition of particle-associated contaminants. Both approaches have been proved to identify unambiguously tsunamites in sedimentary archives and to reveal detailed insights into the tsunami-driven dispersion of particle-associated organic matter. Generally, the organic geochemical proxies as tested in this study can be reliably used to identify and characterize tsunami deposits in the sedimentary record. Finally, this strategy can be transferred to other locations affected by tsunamis for an in-depth characterization of the destruction and environmental changes induced by tsunami events.

**Supplementary information:**

The online version contains supplementary material available at 10.1007/s10661-022-09989-4.

## Introduction

Natural hazards with subsequent environmental pollution are important issues in today’s world (Depietri, 2020). Notably, natural disasters with a release of pollutants have occurred more frequently in recent decades (Huppert & Sparks, 2006). Some of the most devastating natural hazards are earthquakes followed by tsunamis, causing widespread damage to coastlines and great loss of infrastructures and human lives (Wirtz et al., 2014). Destructive tsunamis show the importance of a fundamental comprehension of event processes such as hydrodynamics, effects of topography and sedimentation. Understanding destruction- and erosion-connected origin, release, transportation, distribution and deposition of pollutants and natural substances is an important parameter of tsunami research.

The Pacific Ocean with its surrounding Ring of Fire is the most tsunami-prone area in the world (Hinga, 2015). High seismic activity emanates for instance from the Japan Trench off northeastern Japan, which is responsible for several earthquake-induced tsunamis in the last centuries: AD 1896 Meiji-Sanriku, 1933 Showa-Sanriku and 2011 Tohoku-oki tsunamis (Goto et al., 2019; Nakamura et al., 2012). The 2011 Tohoku-oki earthquake (M_w_ 9.0) with the eponymous tsunami is particularly noteworthy (Tsuji et al., 2014) due to the proximity to the densely populated and industrialized coastal areas of Japan (Hinrichsen, 1999). The northeastern prefectures, Aomori, Iwate, Miyagi and Fukushima, were all severely affected by the tsunami (International Research Institute of Disaster Science, 2013). The impact, erosion and destruction caused by the tsunami released not only different natural geochemical substances but also numerous pollutants (Bellanova et al., 2020). However, the efforts for rapid cleanup have removed tsunami sediment archives (Bellanova et al., 2020; Chagué-Goff et al., 2017), but the 2011 tsunami deposits in the northernmost region (Aomori prefecture) remain well-preserved and accessible to study the effects and distribution of organic geochemical markers of the tsunami.

Several organic geochemical studies provided the potential for suitable organic indicators of natural and anthropogenic origin that can act as a marker of tsunami impact. Natural substances were sporadically used as indicator compounds for a marine influence on tsunami sediments (Alpar et al., 2012; Mathes-Schmidt et al., 2013; Shinozaki et al., 2015; Bellanova et al., 2020). More unspecific aliphatic hydrocarbons (Bellanova et al., 2020; Mathes-Schmidt et al., 2013; Shinozaki et al., 2015) have been used to differentiate tsunami-affected and non-affected sediments. However, the more specific anthropogenic markers have been used only very rarely as tsunami indicators in sedimentary deposits, with only PAHs (Tipmanee et al., 2012; Bellanova et al., 2019, 2020), pesticides and halogens (Bellanova et al., 2020) have been successfully applied.

This study intended to identify and test a suitable set of organic indicators of both anthropogenic and natural origin to characterize the tsunami impact in sedimentary archives along the Aomori coast. In a second step, these indicators are used to point to different processes induced by the tsunami event, comprising e.g. transport and mixing processes of sedimentary material and associated contaminants.

## Material and methods

### Study area

**Fig. 1:**
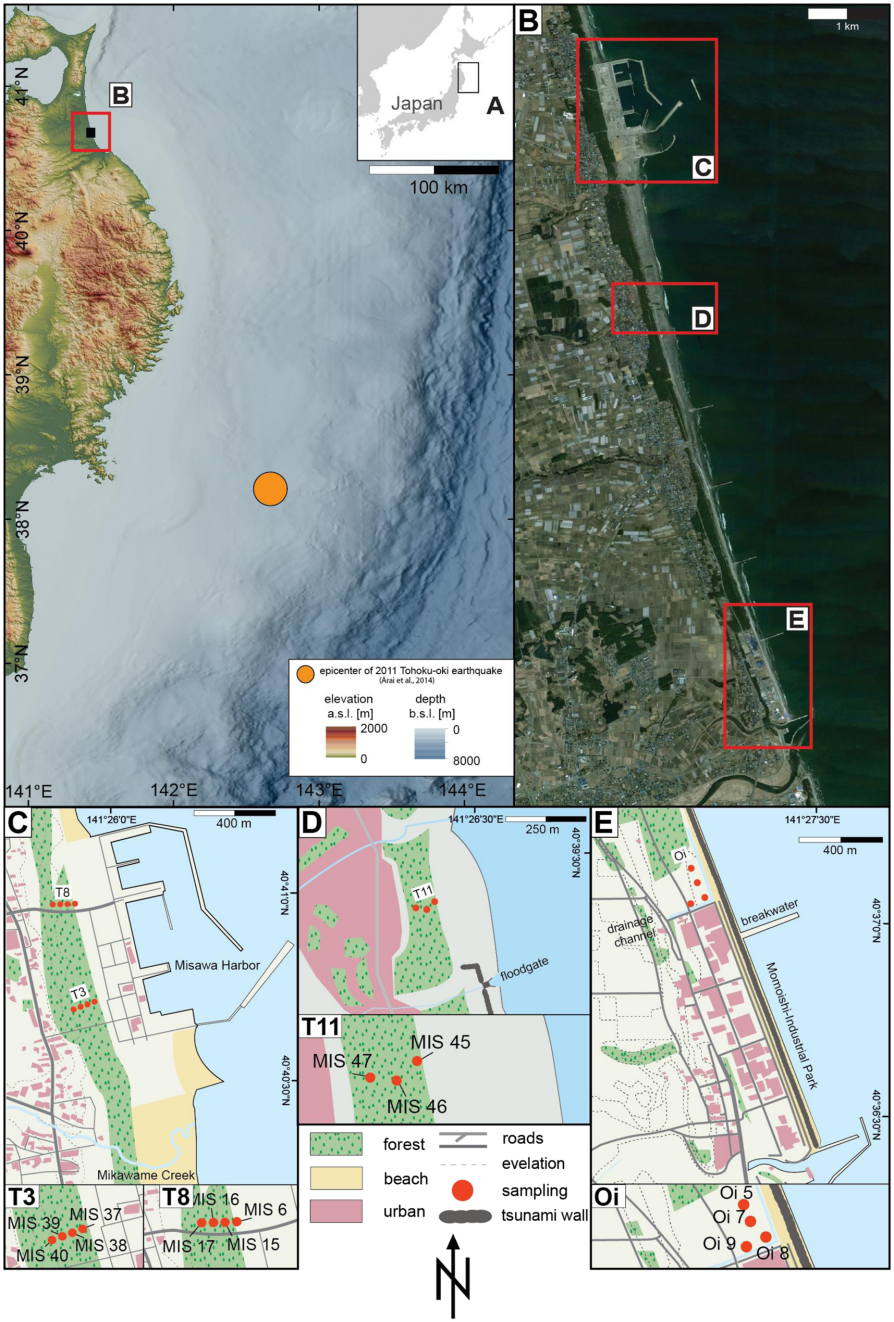
Overview of the study area. **A** Location of the study area with reference to the epicenter of the 2011 Tohoku-oki earthquake (Arai et al., 2014), **B** overview of the three field sites along the Aomori coastline **C** Study area of the Misawa harbor with two transects (T3 and T8) perpendicular to the coast, **D** Futakawame field location with the coastal-perpendicular transect T11, **E** Study area and transect (Oi) north of the Momoishi industrial park (Oirase). Maps are based on open-access SRTM data (USGS EROS data through EarthExplorer; http://earthexplorer.usgs.gov/), processed using ArcGIS Desktop 10.2 (ESRI) and illustrated using Adobe Illustrator (Creative Cloud, https://adobe.com/products/illustrator)

The study area is located within a 10 km stretch between Misawa and Oirase along the northeastern coast of the Aomori Prefecture (Fig. 1). The region is characterized by a 100–200 m wide coastal lowland with a coastal parallel dune ridge and coastal-perpendicular 100 m long breakwaters every kilometer (Nakamura et al., 2012; Tanigawa et al., 2018). A control forest consisting of black pines (*Pinus thunbergii*) covers the coastal lowland and continues on further inland. The lowland is delimitated by a 10–20 m high terrace scarp (Nakamura et al., 2012; Nandasena et al., 2012). Several rivers and channels, such as the Misawa River, Ino River and Mikawame creek, cross the coastal area and drain into the Pacific Ocean.

The study area further subdivides into three main target areas: (I) the Misawa harbor (northern location), (II) Futakawame (central location), and (III) the Momoishi-Industrial Park near Oirase (southern location; Fig. 1). The northernmost location of the Misawa harbor includes the harbor infrastructure, three piers with upstream breakwaters, and a recreational beach. The central target area Futakawame is located south of the Mikawame creek. This location is characterized by a more rural environment with an approx. 150-m-wide beach, sparsely vegetated dune ridges and the inland adjacent coastal control forest (Fig. 1). In the backcountry, a residential area is located, and two irrigation channels discharge into the Pacific Ocean.

The Momoishi-Industrial Park (Oirase) is located about 10 km south of the Misawa harbor. The industrial area is separated from the ocean only by a approx. 4–5-m-high seawall (Fig. 1). Local production facilities include food packaging, rental companies and steel construction companies. North of the industrial complex is a fallow area with sparse vegetation and a small sewage channel.

Along the targeted Aomori coastline, approx. 320 km from the 2011 Tohoku-oki earthquake epicenter (Nakamura et al., 2012), at least three waves were reported of which the second one was the strongest with estimated run-up heights of 10.9 m (Koiwa et al., 2014). Buildings were flooded and (partially) destroyed, a seawall was heavily damaged, oil tanks were tipped over, and ships, barrels, buoys and fishing nets were transported inland (Misawa City Board of Education, 2012; Nakamura et al., 2012; Nandasena et al., 2012; Hazarika et al., 2013). The tsunami inundation at Mikawame creek (southern edge of the Misawa harbor) was very extensive, reaching 550 m inland, where it destroyed parts of the residential area. The harbor area was covered with sediments (Hazarika et al., 2013) and run-up heights of 5.8 m were measured (Nakamura et al., 2012). At the Futakawame site, eyewitnesses reported that the tsunami flooded the residential area inland of the forest and demolished several buildings, including a pig farm. Also due to local topographical conditions, the two streams have overflowed their banks and parts of the seawall system were destroyed, including a floodgate (Tanigawa et al., 2018). At the southernmost location Oirase, the tsunami inundated and devastated most of the industrial park. Reports and field surveys after the event have summarized and published the impact on the region in Misawa and Oirase. In Misawa, 6 km^2^ of land and 169 buildings and Oirase City reported 3 km^2^ with 213 demolished buildings were affected (National Institute for Land and Infrastructure Management, 2011; International Research Institute of Disaster Science, 2013).

### Sample material

**Fig. 2:**
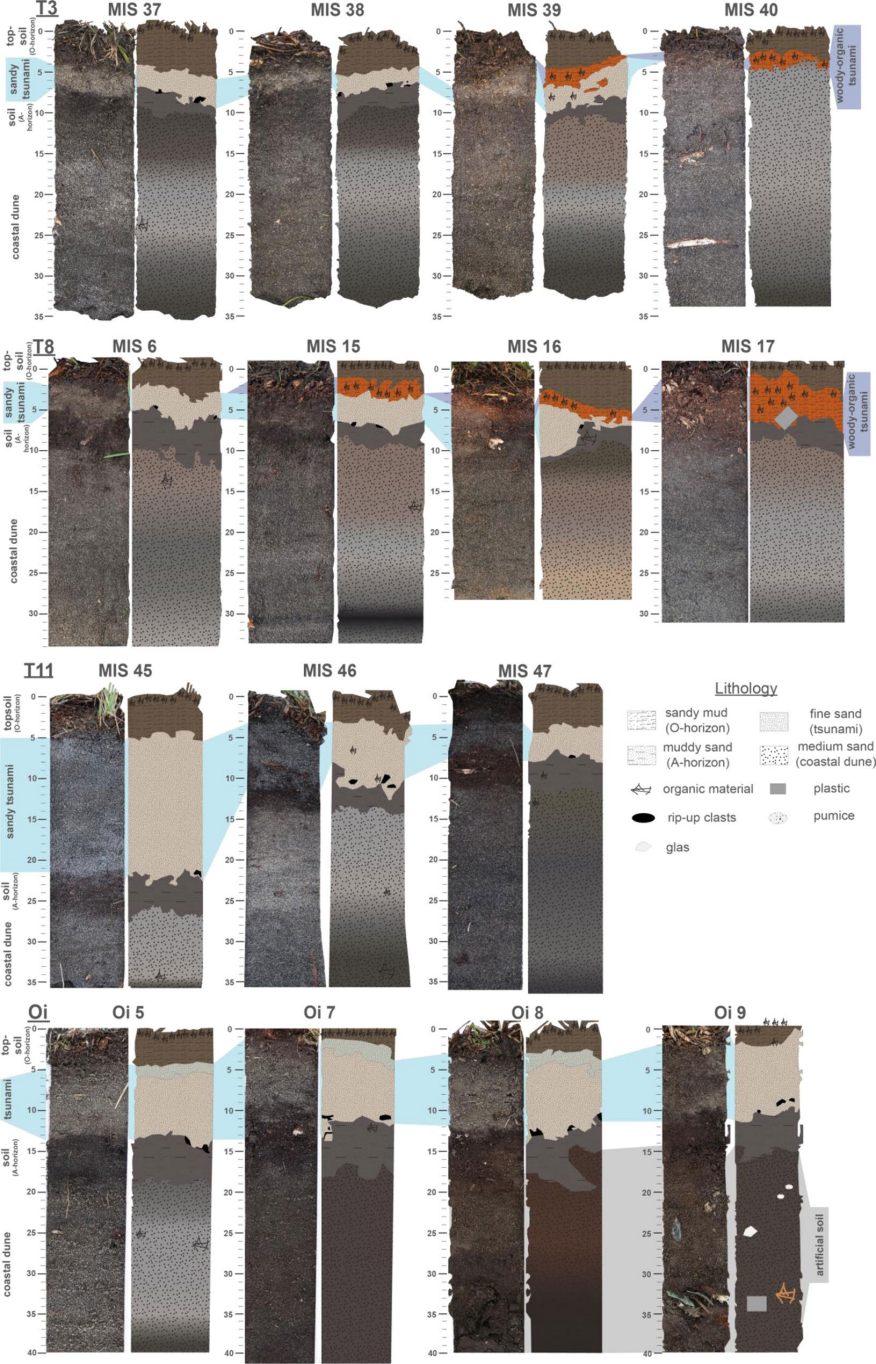
Stratigraphic profiles and sediment profile photographs of all 15 geoslicer. Sediment profiles indicate the four respectively five stratigraphic units, the thinning inland of the 2011 Tohoku-oki tsunami deposit (T3, T8 and T11), transition of the sandy tsunami layer to the non-sandy tsunami layer (T3 and T8). Figure was illustrated using Adobe Illustrator

Samples originate from a field survey in November 2018 along the Misawa coastline. Fifteen sediment cores from four coastal transects (Fig. 1), with three coastal perpendicular transects, were obtained by using a GeoSlicer (Fig. 2). Each sediment profile reaches approx. 40 cm depth. A total of 61 samples (between 7 and 130 g) from tsunami deposits, pre- and post-tsunami soils and dune sediments were collected in pre-cleaned aluminum-coated containers and stored at 4–7 °C to avoid cross-contamination and microbial alterations.

Transects T3 and T8 in the harbor area are located within the coastal control forest and are comprised of each of four sediment profiles (MIS 6-MIS 17 and MIS 37-MIS 40) from ca. 275–350 m inland (Figs. 1 and 2, Table 2-appendix). Transect T11, located at the central Futakawame location, consists of three sediment profiles (MIS 45-MIS 47) ca. 190–240 m inland (Figs. 1 and 2, Table 2-appendix). The Oirase transect consists of 4 sediment profiles taken all in similar distances to the coast (55–70 m) but with varying distances to the Momoishi-Industrial Park, with Oi 5 being located furthest apart from the industrial complex, Oi 7 being located in the center and two sediment profiles (Oi 8 and Oi 9) in close proximity to the industry (Figs. 1 and 2, Table 2-appendix). For elevation and coordinates of each core, see Table 2-appendix.

### Total organic carbon

For total organic carbon (TOC) measurements, bulk samples were prepared by the removal of macro plant residues (e.g., needles and leaves), drying and subsequent homogenization. Of each sample, 100 mg ground material was measured using the Elementar LiquiTOC II (Elementar Analysensysteme GmbH, Germany). Duplicate measurements were conducted, and the relative standard deviation ranged from 0.1 to 1.5%.

### Extraction and fractionation

Extraction of all samples was performed by solid–liquid extraction via overhead shaking with 110 mL of acetone (twice, for 4 and 24 h, respectively) and 110 mL of *n*-hexane (24 h) in an Erlenmeyer flask as described in detail by Bellanova et al. (2019). The combined extracts were collected in a separatory funnel and the aqueous phase was removed. The raw extract was reduced via rotary evaporation (at 300 mbar) to approx. 1 mL and dried over anhydrous granulated sodium sulfate (Na_2_SO_4_). Each extract was desulphurized by adding activated copper powder in ultrasonic agitation.

The raw extracts were fractionated into six fractions using column chromatography on a micro-column with 2 g activated silica gel (40 µm, 60 Å, J.T. Baker^™^) and different eluent mixtures of *n*-pentane, dichloromethane and methanol (according to Schwarzbauer et al., 2000). To each individual fraction, 50 μL of internal surrogate standard solution was added (5.8 ng/μL fluoroacetophenone, 6.28 ng/μL d_10_-benzophenone and 6.03 ng/μL d_34_-hexadecane). The fractions were reduced to an analytical volume (20–200 µL) for GC–MS analysis.

### Gas chromatography-mass-spectrometry analysis

The gas chromatography-mass-spectrometry (GC–MS) analysis was performed on a quadrupole GC–MS system (Thermo Finnigan Trace GC/MS, Thermo Finnigan LLC, USA) with helium as carrier gas. The GC–MS was operating in full scan mode ranging from 35 m/z to -500 m/z in positive electron impact ionization mode (EI +) with 7 eV electron energy. For the analysis, 1 μL of each fraction was injected into the GC–MS at a start temperature of 60 °C with a splitless time of 60 s. The start temperature was held for 3 min, followed by a temperature ramp of 3 °C/min to 310 °C with a final isothermal time of 20 min.

Identification of the organic compounds was achieved by comparing mass spectra and gas chromatographic retention with those of mass spectral libraries (NIST 2014 database National Institute of Standards and Technology – US Department of Commerce, USA) and published data (e.g., Schwarzbauer, 2006 and references therein; Chen et al., 2016).

Standard reference material was used for verification and quantification. Quantification was based on the integration of specific ion chromatograms using XCalibur^™^ (Thermo Fisher Scientific Inc., USA) and external four-point calibrations. Inaccuracies during measurements, volume reduction and retention times were corrected by the surrogate standard (Dsikowitzky et al., 2004). Each detected compound was quantified with an individual m/z value distinct for the compound group. Additionally, all samples were normalized to TOC (ng/g_TOC_) and to the fine grain fraction (< 0.063 mm) (ng/g_FGF_) to obtain comparable results.

### Grain size analysis

For grain size analysis, duplicate samples (10–20 g) were taken, that previous alteration by geochemical processes is not given. All samples were dried at 40 °C to constant mass and sieved to < 2 mm. Organic matter in the samples was removed with hydrogen peroxide (H_2_O_2_, 15%) and sodium pyrophosphate (Na_4_P_2_O_7_ (0.1 mol*l^−1^))) was added to limit coagulation (Gee & Or, 2002). Grain size measurements of grains < 2 mm (0.5–2.5 g) were performed with a laser diffraction particle size analyzer (Beckman CoulterTM LS13 320 with additional PIDS technology, Beckman Coulter GmbH, Germany) based on Schulte and Lehmkuhl (2018). Duplicate measurements were made to minimize the error margin and the mean results are in the range of 0.04–2000 μm. For statistical analysis, the GRADISTAT V8 software after Blott and Pye (2001) was used to calculate the granulometric parameter (Fig. S1-appendix) based on Folk and Ward (1957).

## Results and discussion

### Sedimentology

For the identification and determination of the 2011 Tohoku-oki tsunami layer a sedimentological investigation of the sedimentary archive at the Aomori coast has been performed. The sedimentological properties of the investigated sediment cores are illustrated in Fig. 2. Subsamples for further geochemical investigations were taken from six different stratigraphic units based on the field observation and the grain size analysis: topsoil (organic surface layer/O-horizon), 2011 tsunami deposit (sandy), 2011 tsunami deposit (woody-organic), underlying pre-2011-soil (A-horizon), coastal paleodune deposits and artificial backfill material (see Fig. 2). In more detail, the lowermost unit of a coastal palaeodune consists of a podzol and is characterized by medium sand with inclusions of heavy minerals and a color variation from brownish to dark greyish. The dunes have undergone changes, in which pine trees have been planted to protect the residential and agricultural areas in the hinterland from salt spray and tsunami damage as described by Tanigawa et al. (2018). The palaeodune deposits are not visible in all sediment profiles of Oirase), but artificial backfilled material at identical depth, distinguished by medium brown sand with residues of plastic, glass and wood (see Fig. 2, Oi5 to Oi9). The second stratigraphic unit is the pre-2011-soil, which is an A-horizon of a dark brown, silty sand with a high content of organic material. The layers of interest for this study are the 2011 Tohoku-oki tsunami deposits as marked in blue in Fig. 2. Characteristic for the sandy tsunami layer is mainly the light-colored fine sand with a sharp contact to the underlying pre-2011-soil. These findings are consistent with observations by Nakamura et al. (2012) and Koiwa et al. (2014), who report sharp contact from the 2011 tsunami layer to the underlying pre-2011 soil along the Aomori coast near Misawa. The sudden change and distinct differentiation in grain size and color to the surrounding layers suggest a mixture of allochthonous material. Inland thinning is observed, typical for tsunami deposits, for instance in transect T11 (from 16 cm thickness in MIS 45 to 3 cm in MIS 47, see Fig. 2). Furthermore, an inland fining of the grain size was detected in most of the transects and rip-up clasts are evident in some profiles (Fig. 2). Both observations of inland thinning and fining of the tsunami layers and the detected rip-up clasts are consistent with previous studies in the region (Nakamura et al., 2012; Koiwa et al., 2014; Tanigawa et al., 2018; Bellanova et al., 2021). With the inland thinning out and ultimately disappearance of the sandy tsunami layer (approx. 300 m in T3 and T8), a light brown layer starts to appear on top of the tsunami sand. This layer is characterized by organic-rich woody material and is interpreted by Bellanova et al. (2021) as a non-sandy, woody-organic tsunami deposit, which is transported further inland during the tsunami inundation and was hardly affected by backwash currents due to low flow energy further inland. The uppermost unit, the organic surface layer with a low mineral content (topsoil/O-horizon), is characterized by organic-rich, dark brownish fine-grained material with residues of needles and other plant remains.

### Normalization

**Fig. 3:**
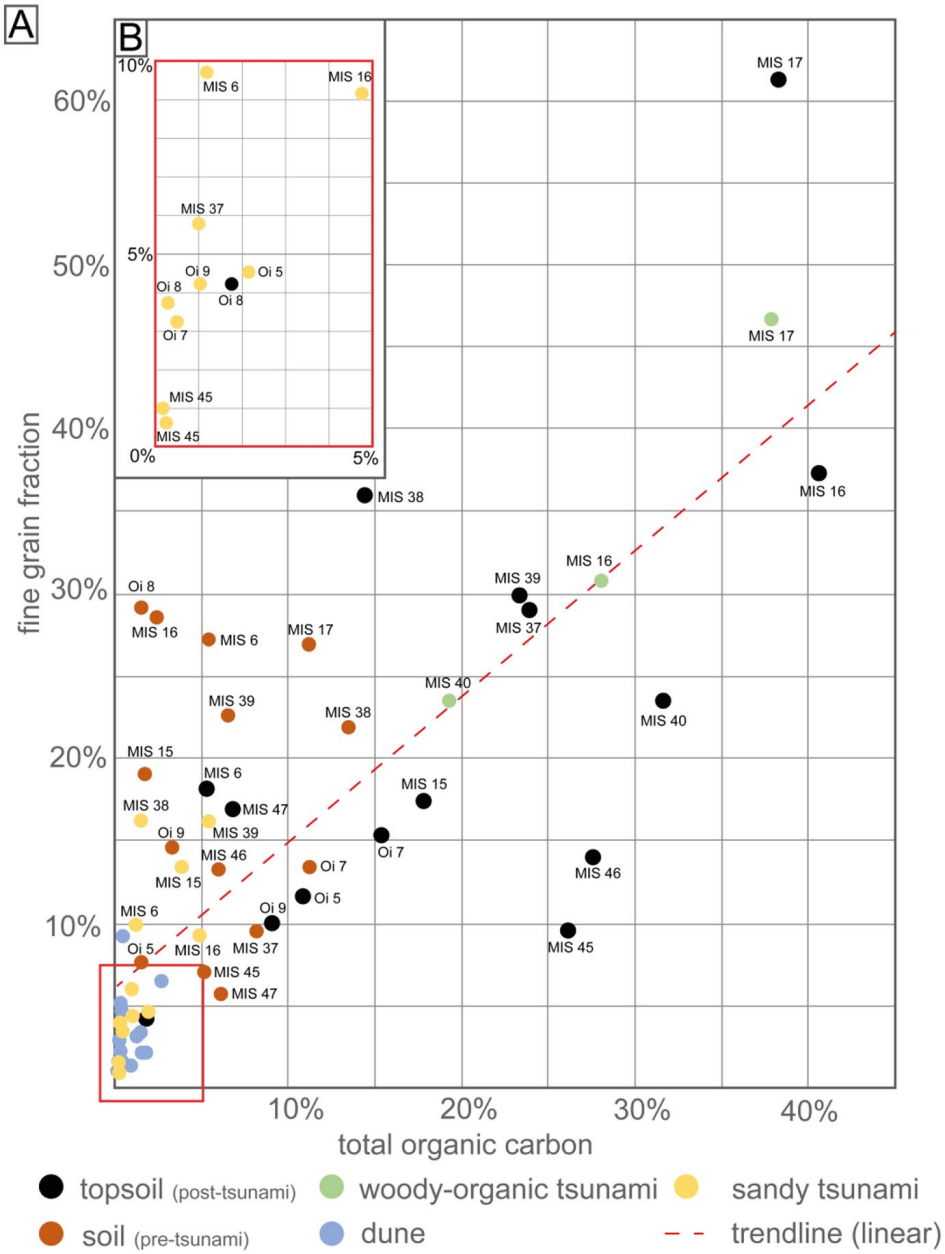
Comparison of TOC and fines grain fraction **A** all samples with sediment profile labels **B** closer view of tsunami and paleodune samples with lower values. Data were plotted by using Microsoft Excel 365 and illustrated using Adobe Illustrator

In this study, TOC values as well as grain size distribution have been measured for all analyzed sediment layers. The results are given in Table 1 and illustrated in Figs. 3 and 13. TOC values covered a wide range between 0.1 and 40%, and fine grain fractions (FGF < 0.063 mm) varied highly between approx. 1 and 60%.

**Table 1:** Concentration and ratios of all detected compounds (TOC-normalization). Data were plotted by using Microsoft Excel 365

	**Concentrations in ng/g**_**TOC**_				**Marine vs. terrestrial approach**			**Petrogenic parameter**								**Sewage-derived marker**		**Old burden parameter**	
	**Core**	**Description**	**Depth [cm]**	**TOC [%]**	***n*-Alkane C**_**12**_**-C**_**32**_	**TAR**	***n*-Aldehyde C**_**20**_**-C**_**32**_	**OEP**	**Hopane**	**22S/(22S + 22R) C**_**31**_	**22S/(22S + 22R) C**_**32**_	**PAHs (parent)**	**PAHs (alkylated)**	**A/(A + P)**	**F/(F + Py)**	**LABs**	**DIPNs**	**DDX**	**PCBs**
**transect T3**	MIS 37	Topsoil	1–4	24.10	280,000	32	9200	3.2	1700	0.6	0.6	61,000	6800	0.04	0.6	590.0	13	37	28
	MIS 37	Tsunami	5–8	1.02	1,900,000	4.3	13,000	2.4	35,000	0.6	0.6	2,400,000	160,000	0.01	0.7	6600.0	200	750	1100
	MIS 37	Pre-2011 soil	8–10	8.22	260,000	10	25,000	2.7	1200	0.5	0.6	39,000	12,000	0.02	0.7	370.0	35	220	190
	MIS 37	Coastal dune	11–14	0.23	1,600,000	29	71,000	3.3	2200	0.5	0.6	28,000	2800	0.00	0.8	5900.0	270	1300	390
	MIS 37	Coastal dune	18–24	0.10	1,200,000	32	16,000	5.4	67	< LOQ	1.0	230,000	28,000	0.03	0.6	76,000.0	3800	1100	7500
	MIS 38	Topsoil	2–4	14.48	320,000	11	17,000	4.4	5000	0.6	0.6	38,000	8200	0.11	0.5	590.0	22	84	13
	MIS 38	Tsunami	4–6	1.50	1,300,000	4.6	31,000	2.8	16,000	0.6	0.6	540,000	130,000	0.10	0.6	2900.0	52	380	240
	MIS 38	Pre-2011 soil	7–10	13.51	55,000	6.2	2700	5.2	390	0.6	0.6	32,000	2500	0.00	0.5	93.0	5.8	190	31
	MIS 38	Coastal dune	14–18	0.30	350,000	13	< LOQ	4.2	1100	0.6	0.7	56,000	8300	0.00	0.6	< LOQ	< LOQ	340	< LOQ
	MIS 39	Topsoil	0–5.5	23.46	100,000	4.1	24,000	3.6	1400	0.7	1.0	24,000	6200	0.06	0.6	370.0	9.6	54	22
	MIS 39	Tsunami	6–8.5	5.43	300,000	2.6	40,000	2.6	4000	0.6	0.8	310,000	71,000	0.03	0.7	850.0	43	380	93
	MIS 39	Pre-2011 soil	9–12	6.57	940,000	4.7	76,000	6.2	6700	0.6	1.0	87,000	25,000	0.07	0.6	830.0	40	430	82
	MIS 39	Coastal dune	15–18	1.68	1,400,000	32	32,000	4.8	3600	0.7	1.0	160,000	30,000	0.04	0.5	980.0	52	830	98
	MIS 40	Topsoil	0–2	31.68	340,000	7.6	64,000	4.2	5600	0.8	1.0	31,000	2500	0.92	0.0	670.0	33	81	59
	MIS 40	Woody-organic tsunami	2–4	19.30	250,000	6.5	19,000	3.1	3300	0.8	1.0	100,000	22,000	0.06	0.5	1800.0	27	73	4.1
	MIS 40	Coastal dune top	6–9	2.78	1,100,000	33	92,000	2.9	17,000	0.6	1.0	120,000	30,000	0.03	0.5	1300.0	130	290	98
	MIS 40	Coastal dune bottom	31–36	0.05	1,200,000	17	120,000	7.3	4700	0.3	0.8	240,000	43,000	0.04	0.6	41,000.0	3100	350	< LOQ
**transect T8**	MIS 6	Topsoil	0–2	5.37	860,000	9.4	64,000	3.0	4300	0.7	0.9	430,000	69,000	0.03	0.5	1100.0	0.1	< LOQ	< LOQ
	MIS 6	Tsunami	2–5	1.22	220,000	7.0	180,000	2.2	3400	0.6	1.0	290,000	31,000	0.03	0.5	1800.0	550	73	67
	MIS 6	Pre-2011 soil	5–9	5.43	91,000	10	15,000	5.9	490	0.6	0.6	7600	1700	0.03	0.6	360.0	72	160	35
	MIS 6	Coastal dune	12–15	0.25	4,100,000	60	1400	2.9	1200	0.6	0.6	39,000	5500	0.02	0.6	5500.0	1400	170	67
	MIS 15	Topsoil	1–4	17.86	50,000	7.3	46	3.0	380	0.6	0.7	5900	1200	0.04	0.5	78.0	3.1	17	4.6
	MIS 15	Tsunami	4.5–6	3.93	440,000	6.4	16,000	2.1	2300	0.7	0.6	22,000	1400	0.07	0.4	480.0	12	29	15
	MIS 15	Pre-2011 soil	6–9	1.78	1,900,000	14	170,000	3.7	4400	0.7	0.8	130,000	31,000	0.02	0.6	4300.0	350	2300	140
	MIS 15	Coastal dune	18–20	0.10	200,000	23	2900	5.4	490	0.6	0.9	26,000	4500	0.02	0.5	1800.0	140	52	4.1
	MIS 16	Topsoil	0–3	40.61	240,000	14	27,000	2.0	450	0.7	0.9	27,000	4700	0.09	0.5	740.0	23	0.1	29
	MIS 16	Woody-organic tsunami	2–5	28.07	34,000	9.1	24,000	1.3	380	0.7	1.0	3600	930	0.06	0.6	260.0	100	23	13
	MIS 16	Tsunami	4–6	4.94	430,000	9.0	48,000	2.0	3200	0.5	0.9	26,000	5400	0.05	0.6	2800.0	1300	0.1	86
	MIS 16	Pre-2011 soil	7–9	2.46	460,000	12	160,000	3.0	2200	0.6	0.9	74,000	13,000	0.02	0.6	670.0	10	530	46
	MIS 17	Topsoil	0 -2	38.27	500,000	11	67,000	2.5	5700	0.8	1.0	27,000	3800	0.03	0.6	520.0	17	46	15
	MIS 17	Woody-organic tsunami	2–5.5	37.85	110,000	6.1	10,000	2.5	890	0.7	1.0	17,000	3800	0.02	0.6	200.0	1.4	28	1.9
	MIS 17	Pre-2011 soil	7–9	11.26	340,000	7.3	27,000	2.7	2900	0.5	1.0	34,000	9300	0.01	0.6	450.0	7.3	380	19
	MIS 17	Coastal dune	12–15	1.47	220,000	7.0	1700	2.9	720	0.4	1.0	72,000	5600	0.01	0.7	1200.0	240	80	130
	MIS 17	Coastal dune	24–27	0.08	75,000	8.4	< LOQ	3.6	540	0.4	0.7	8000	2600	0.27	0.6	1300.0	220	< LOQ	20
**transect T11**	MIS 45	Topsoil	0–1	26.14	53,000	2.9	11,000	3.8	9	0.5	0.5	2900	890	0.09	0.6	6200.0	230	64	93
	MIS 45	Topsoil	2–4.5	0.22	280,000	6.6	220,000	2.1	290	0.6	0.7	32,000	7500	0.17	0.5	3300.0	280	250	58
	MIS 45	Tsunami	7.5–9.5	0.08	2,400,000	10	710,000	2.2	5300	0.6	0.7	140,000	27,000	0.18	0.5	16,000.0	890	1000	280
	MIS 45	Tsunami	13–18	0.10	3,300,000	27	200,000	2.8	3300	0.6	0.7	32,000	6500	0.38	0.6	11,000.0	580	320	95
	MIS 45	Pre-2011 soil	21–26	5.25	390,000	9.5	140,000	2.0	620	0.7	0.9	29,000	5200	0.09	0.7	450.0	58	0.01	120
	MIS 45	Coastal dune	26–29.5	0.83	1,800,000	20	460,000	1.8	4900	0.6	0.9	550,000	160,000	0.09	0.5	2300.0	250	1700	370
	MIS 46	Topsoil	0–4	27.60	720,000	23	40,000	6.6	100	0.6	0.7	3400	880	0.02	0.6	1200.0	21	100	19
	MIS 46	Tsunami	7–10	0.27	1,100,000	18	240,000	2.0	2200	0.6	0.7	96,000	19,000	0.04	0.5	4000.0	200	500	91
	MIS 46	Pre-2011 soil	12–16	6.05	220,000	13	64,000	2.3	520	0.6	0.8	20,000	3900	0.13	0.6	230.0	21	320	54
	MIS 47	Topsoil	0–3.5	6.84	210,000	1.7	72,000	3.7	830	0.6	0.6	49,000	13,000	0.03	0.6	1500.0	89	130	410
	MIS 47	Tsunami	3.5–5	0.35	2,200,000	11	350,000	2.1	4800	0.6	0.7	110,000	24,000	0.16	0.6	7100.0	640	1800	380
	MIS 47	Pre-2011 soil	7–11	6.21	1,000,000	17	140,000	4.3	270	0.7	0.9	23,000	4700	0.11	0.7	2700.0	62	910	110
**transect Oi**	Oi 5	Topsoil	0–4	10.87	1,300,000	33	40,000	2.5	940	0.6	0.8	3500	910	0.05	0.6	3100.0	13	< LOQ	2.5
	Oi 5	Tsunami	4–6	2.03	260,000	2.0	17,000	2.0	1900	0.6	0.6	11,000	1200	0.01	0.5	5500.0	55	< LOQ	160
	Oi 5	Pre-2011 soil	13–18	1.59	6,200,000	13	81,000	2.0	18,000	0.6	0.7	11,000	2800	0.04	0.6	950.0	29	< LOQ	170
	Oi 5	Coastal dune	21–25	0.39	320,000	16	1800	1.8	430	0.5	0.6	26,000	2700	0.02	0.6	2200.0	48	< LOQ	59
	Oi 7	Pre-2011 soil	0 -2	15.45	680,000	18	42,000	1.7	340	0.7	0.8	7900	820	0.01	0.6	750.0	15	< LOQ	39
	Oi 7	Tsunami	4–7	0.47	1,000,000	2	110,000	1.5	4500	0.6	0.6	60,000	20,000	0.09	0.5	8600.0	340	190	860
	Oi 7	Pre-2011 soil	13–17	11.21	170,000	21	16,000	2.3	390	0.6	0.8	4300	1200	0.02	0.6	260.0	12	7.0	72
	Oi 8	Topsoil	0–4	1.89	4,300,000	22	11,000	3.5	14,000	0.6	0.6	7100	2100	0.07	0.5	510.0	53	< LOQ	50
	Oi 8	Tsunami	8–13	0.29	2,000,000	4.6	330,000	1.9	18,000	0.6	0.6	57,000	23,000	0.08	0.5	4200.0	47	400	320
	Oi 8	Pre-2011 soil	13–17	1.58	73,000	1.2	20,000	5.4	60	0.6	0.6	4500	1500	0.04	0.5	260.0	17	63	40
	Oi 8	Artificial	22–27	0.39	720,000	0.2	79,000	3.5	700	0.6	0.6	520,000	160,000	0.08	0.5	5700.0	290	1600	2900
	Oi 9	Topsoil	0–2	9.12	1,500,000	14	28,000	3.8	3800	0.5	0.8	1500	4400	0.28	0.2	780.0	41	< LOQ	46
	Oi 9	Tsunami	6–8	1.04	1,700,000	5.3	630,000	2.2	15,000	0.6	0.6	350,000	80,000	0.02	0.5	2300.0	100	60	560
	Oi 9	Pre-2011 soil	10–14	3.34	1,100,000	11	380,000	3.3	2400	0.6	0.7	49,000	7900	0.01	0.9	900.0	91	0.1	410
	Oi 9	Artificial	18–25	0.41	1,500,000	0.5	21,000	1.7	1700	0.6	0.6	520,000	76,000	0.01	0.4	9100.0	87	< LOQ	5400

∑PAH_29_: naphthalene; acenaphthylene; biphenyl; acenaphthene; fluorene; dibenzofuran; phenanthrene; anthracene; fluoranthene; pyrene; benzo[ghi]fluoranthene; benzo[c]phenanthrene; benz[a]anthracene; chrysene/triphenylene; cyclopenta[cd]pyrene; benzo[b]fluoranthene; benzo[k]fluoranthene; benzo[j]fluoranthene; benzo[e]pyrene; benzo[a]pyrene; perylene; dibenz[a,j]anthracene; indeno[1,2,3-cd]pyrene; dibenz[a,c]anthracene; dibenz[a,h]anthracene; benzo[b]chrysene; picene; benzo[ghi]perylene; anthanthrene

∑aPAH_10_: methylanthracenes(-phenanthrenes); dimethylanthracenes(-phenanthrenes); methylpyrenes(-fluoranthenes); dimethylpyrenes(-fluoranthenes); methylbenzanthracenes(-phenanthrenes); dimethylbenzo(a)anthracenes(-phenanthrenes); methylbenzo(a)pyrenes(-fluoranthenes); methylbenzpyrenes(-fluoranthenes); methylnaphthalense; dimethylnaphthalenes

Both bulk parameters represent basic determinants for normalization of heterogenous sediment samples to ensure comparability of organic-geochemical values amongst them. This is based on a preferred accumulation of organic substances on fine grain-sized particles (mud and silt) and in organic-rich particulate matter (e.g. Jeng, 2007; Müller et al., 2000; van Kaam-Peters et al., 1998; Wilcke et al., 1997). Normalization of results to TOC has been used as a standard technique in both, environmental studies (e.g., Prahl & Carpenter, 1987; Meyers, 2003; Commendatore et al., 2012; Schwarzbauer et al., 2018) and in geochemistry-focused tsunami studies (e.g., Bellanova et al., 2019, 2020). In this study, both parameters, TOC values and fine grain fraction, were considered for normalization of the miscellaneous layers (O-horizon, tsunami sand, A-horizon, and paleodune) and their applicability was compared.

Based on past observation (e.g., by Chagué-Goff et al., 2012; Mathes-Schmidt et al., 2013; Wittkopp et al., 2011) it is to be assumed that the tsunami incorporates both marine organic and terrestrial organic material on its way inland, causing the resulting (sandy) tsunami layers to have higher TOC concentrations than normal sand layers (e.g., dunes, beaches). Due to the inland increasing uptake of terrestrial organic material during tsunami inundation, there is also an increase in TOC content the further inland the tsunami sediment is located. Inland increasing organic debris in the tsunami deposits was described for Misawa (Nakamura et al., 2012) and the observation of a woody-organic tsunami layer further supports this (Bellanova et al., 2021). While the tsunami deposits show for a sandy deposit relatively high TOC concentration (up to ca. 5%, Table 1), the surrounding sediments (topsoil – O-horizon and pre-2011-soil—A horizon) exceed these with significantly higher TOC values as they are in a state of soil formation containing higher amounts of humus. Particularly outstanding are the TOC values of the topsoil (O-horizon) of up to 40% (Table 1). Characteristics of the forest soil’s first centimeters are *inter alia* high proportions of micro plant residues or debris accompanied with an underrepresented proportion of mineral components (Blume et al., 2010). Some studies (e.g., De Vos et al., 2005; Lilly et al., 2020; Ostrowska & Porębska, 2012) have reported high organic matter in O-horizons, with comparable values above 40%. In particular, the topsoil samples from MIS 17 and MIS 16, and also the woody-organic tsunami sample of MIS 17, illustrate high TOC values (Fig. 3). Generally, a TOC normalization seems feasible for this study site, although potential difficulties may occur with organic-rich topsoils or O-horizons.

Generally, normalization to grain size is frequently used in environmental studies with a focus on trace and heavy metals (e.g., Aloupi & Angelidis, 2001; Dung et al., 2013; Loring & Rantala, 1992; Roussiez et al., 2005), but it has also been used with respect to organic pollutants. Grain size normalization serves to neglect the effect by grain size variations and subsequent absorption capacities between heterogenous samples (e.g., sandy tsunami layers vs. silty surrounding soils) and to prevent misinterpretation due to elevated accumulation potential in fine grain-sized particulate matter (e.g. Aloupi & Angelidis, 2001; Loring & Rantala, 1992; Wilcke et al., 1996, 1997).

Results from the Aomori coast show, that the samples with a high TOC value also tend to contain a high fine grain content with maximum values of around 60%. This correlation is figured out in Fig. 3 and supports the hypothesis, that TOC and grain size are key controlling factors for the absorption and preservation of organic natural compounds and pollutants in sediments. However, grain size normalization does not allow the consideration of event-related alterations, such as the mixture of different organic-rich particles from marine and terrestrial sources (e.g., erosion and reworking soils, backwash), and their subsequent sedimentation (Chagué-Goff et al., 2012; Wittkopp et al., 2011). This mixture has been documented to cause an increase of the organic matter in sandy tsunami sediments (e.g., Shiki et al., 2008).

Both TOC and fine grain fraction normalizations have their justification, but also each has its limitations. TOC normalization presents constraints in topsoils or O-horizons with high TOCs and undecomposed micro plant residues, therefore misinterpretation of normalized concentrations of topsoils is possible. On the other hand, grain size normalization to the fine grain fraction can only be used to a limited extent as it does not incorporate varying organic carbon contents between different samples. This is especially evident when comparing the variance (on the example of the tsunami deposits) between TOC-normalized samples (5%; Table 1; Fig. 3) and the fine-grain fraction normalized samples (20%; Table S1-appendix) (Fig. 3). TOC normalization presents a more uniform normalization as it eliminates most analytical pitfalls, such as higher organic matter in tsunami layers and differences in absorption capacity of silt and mud. However, the outliers in the TOC content (topsoil of MIS 17 and MIS 16; the non-sandy tsunami layer MIS 17) lead to recurring exceptions in the overall picture of transect T8 in the later course of the results and interpretation. Nonetheless, based on the lower variance, and the tsunami deposits controlling factors the TOC normalization has been selected for normalization as the more suitable way of presenting the obtained results from the Aomori coast (Table 1). Complementarily, grain-size normalized results can be found in the supplementary material (Table S1; Figs. S2-S5-appendix).

### Selection of suitable organic compounds

In order to identify and characterize the tsunami deposits in the sedimentary archives of the Aomori coastline and to evaluate the impact and effects of tsunami on the coastal environment, different environmental- and pollution source-related approaches were applied. This encompassed a wide variety of specific organic compound groups which were detected in the obtained sediment samples. Apart from markers already used in former studies such as PAHs, and alkanes, further indicator classes are applied in this study comprising sewage-derived markers, old burdens and petrogenic substances (hopanes) and tested for suitability in identifying tsunamis.

The marker approach in this study follows two different lines: (i) natural organic substances are considered to distinguish tsunami sediments from the surrounding sediments through concentration shifts and ratios related to their sedimentary sources (marine vs. terrestrial); (ii) anthropogenic marker groups are evaluated as petrogenic parameters, sewage-derived markers and (remobilized) and indicators for old burden contamination, for which an enrichment or accumulation in the tsunami deposits through the destruction, erosion, and secondary remobilization is expected (Bellanova et al., 2020, 2021).

### Natural organic compounds

In the first section, *n*-aldehydes and *n*-alkanes, two environmentally ubiquitous natural components, are used as markers with the capability to differentiate sedimentary origins and properties. Both compound groups were continuously detected in nearly all sediment samples with a high variation of concentrations ranging from 0.05 to 6.2 mg/g_TOC_ for *n*-alkanes or b < LOQ to 710 µg/ g_TOC_ (see Table 1). A distinctive feature of most tsunami sediments is their differing sediment origin, in particular, the differentiation of marine from the terrestrial origin, compared to their surrounding sediments. However, deviating from the general assumption that tsunami deposits are only composed of marine and coastal sediments, tsunami deposits in fact are often comprised also of a substantial amount of terrestrial sediments, which are subject to erosion and redeposition by the tsunami (e.g., Bellanova et al., 2020; Szczuciński et al., 2012). Yet, the hypothesis for a marine vs. terrestrial identification approach is that the tsunami layers contain a stronger marine signature compared to the surrounding more terrestrial-dominated (coastal) soil layers.

The homologous series of *n*-aldehydes (C_9_-C_32_) were identified in all sediment profiles (see Table 1 and Fig. 5). While an exact environmental allocation of the short-chain *n*-aldehydes is not known (Prahl & Pinto, 1987), long-chained *n*-aldehydes (C_20_-C_32_) are introduced into sedimentary systems by land plant waxes (Prahl & Pinto, 1987; Schwarzbauer, 2006). Detected concentrations of *n*-aldehydes reached up to 710,000 ng/g_TOC_ (Table 1). The identified *n*-aldehydes show a strong even- to odd-chained predominance (C_20_-C_32_), which point to an origin of cuticular waxes of higher plants (Goodwin & Mercer, 1983).

Besides the *n*-aldehydes, the homologous series of *n*-alkanes (C_12_-C_35_), were detected in all sediment profiles (see Table 1). These aliphatic hydrocarbons can be distinguished into two classes according to their origin and molecular weight. The short-chain *n*-alkanes (C_12_-C_22_) can be assigned to omnipresent marine and terrestrial biomass production, whereas the long-chain *n*-alkanes (C_23_-C_35_) are an exclusive indicator for terrestrial input by higher land plants (Alpar et al., 2012; Mathes-Schmidt et al., 2013; Schwarzbauer et al., 2018). The total *n*-alkane concentration ranges from 34,000 up to 6200,000 ng/g_TOC_ (Table 1). In addition, the odd-numbered outperform the even-numbered *n*-alkanes and concentration peaks are detectable for C_27_ and C_29_-alkanes. This is an indicator for a dominant input of land plant remains, most likely from black pines in the coastal control forest. For further analysis and evaluation of the short- and long-chain *n*-alkanes, the terrigenous/aquatic ratio (TAR) after Peters et al. (2005) was calculated. The values covered a range between 2.6 and 33, as presented in Table 1. The TAR indicates the source of the aliphatic hydrocarbons based on the ratios of odd-numbered long-chain *n*-alkanes (*n*-C_27_, *n*-C_29_ and *n*-C_31_; Rieley et al., 1991) *versus* the short-chain odd-numbered *n*-alkanes (*n*-C_15_, *n*-C_17_ and *n*-C_19_; Alpar et al., 2012). Higher values of the TAR such as the maximum values in this study around 30 reveal a higher input of higher land plant-derived epicuticular waxes (Bourbonniere & Meyers, 1996; Eglinton & Hamilton, 1963) and the lower the TAR values become the higher the input of marine material (Bellanova et al., 2020; Peters et al., 2005).

**Fig. 4:**
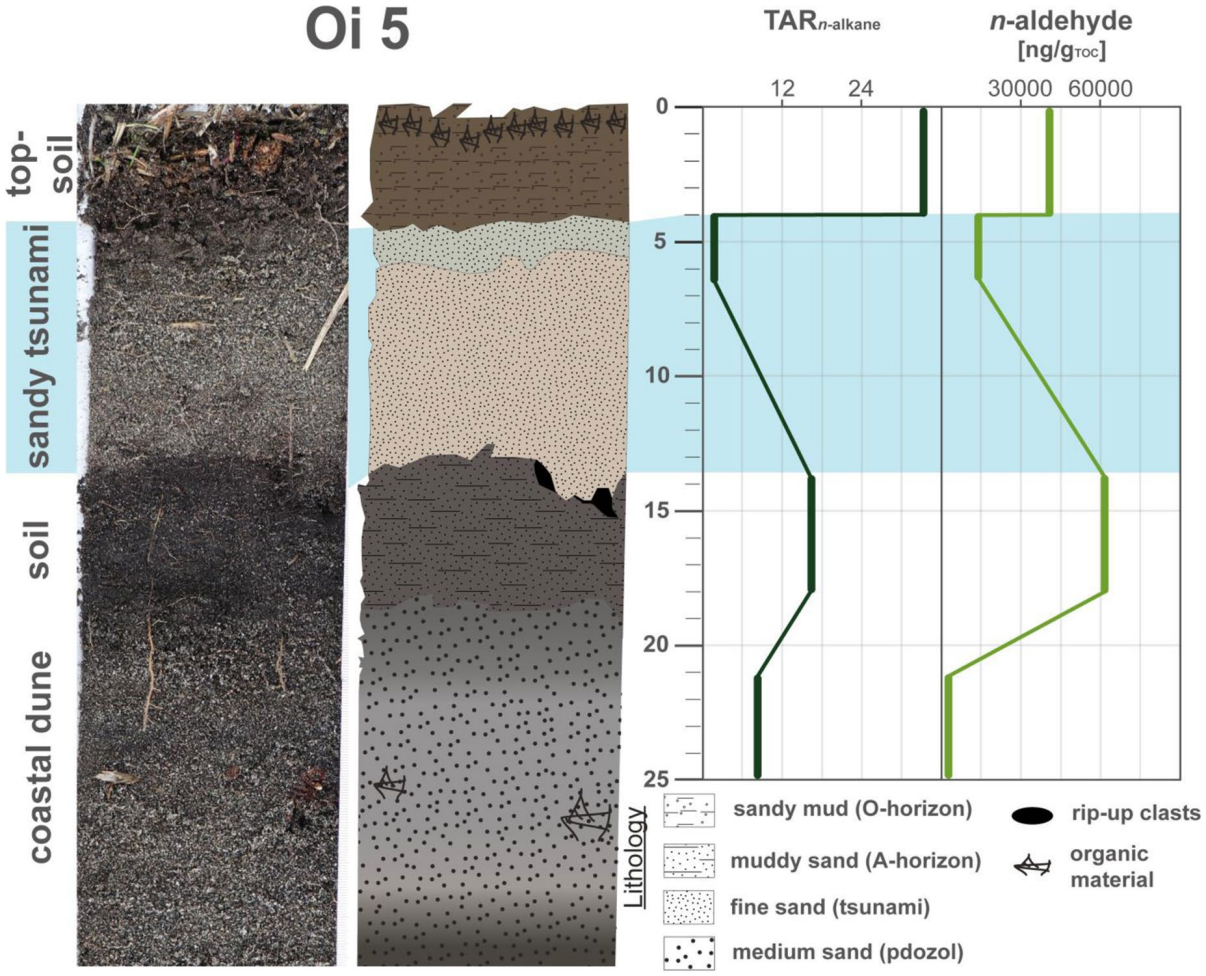
Concentration profiles of the detected natural compounds and ratios, terrigenous/aquatic ratio (TAR—*n*-alkanes ratio) and *n*-aldehydes of Oi 5. Data were plotted by using Microsoft Excel 365 and illustrated using Adobe Illustrator

In sediment profile Oi 5, the usefulness of the marine vs. terrestrial approach is demonstrated by a strong marine dominance of the tsunami deposit, but also a more coastal signature of the paleodune (Fig. 4). The distinctly lower *n*-aldehyde and TAR values of the tsunami deposit in contrast to the surrounding soil layers indicate a marine or marine and terrestrial mixed origin of the tsunami sediments (Fig. 4).

**Fig. 5:**
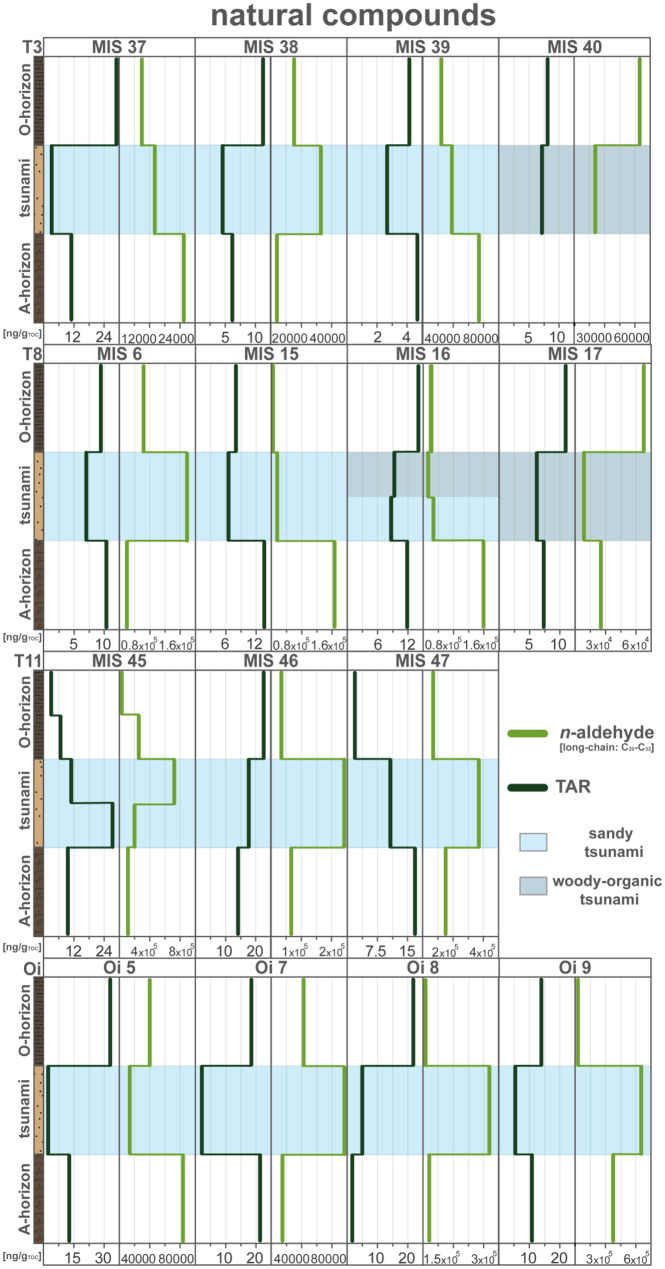
Concentration profiles of the detected natural compounds and ratios, terrigenous/aquatic ratio (TAR—*n*-alkanes ratio) and *n*-aldehydes of all sediment profiles ordered by transects. Figure was illustrated using Adobe Illustrator. Data were plotted by using Microsoft Excel 365 and illustrated using Adobe Illustrator

In contrast to Oi 5, all other tsunami sediments do not indicate such a clear depletion pattern of the long-chain *n*-aldehydes (C_20_-C_32_) but show enrichment of these compounds (Fig. 5). Due to the variation in the long-chain *n*-aldehydes signal, the TAR considered, which reflects a ratio rather than absolute concentrations of substances (Eq. 1). In almost all tsunami deposits (except for transect T11) the TAR presents low values, thus a more dominant marine signal or a decreased terrestrial signal (Fig. 5). By this, location-specific factors are not to be neglected when comparing the different transects. While transects T3, T8 and Oi all show a lower TAR, transect T11 presents a more varying TAR signature.

1 $$TAR=\frac{{n-C}_{27}+ {n-C}_{29}+ {n-C}_{31}}{{n-C}_{15}+ {n-C}_{17}+ {n-C}_{19}} \left(n-\mathrm{alkanes}\right)$$

The pattern deviation of the Futakawame transect (T11), where most tsunami layers are enriched in *n*-aldehydes and indicate varying TAR values, can be linked to its more rural location. More precisely, the beach and sparsely vegetated dunes (Fig. 1) provided a more coastal to the terrestrial environment for sediment uptake during the tsunami land fall. The increased *n*-aldehyde levels could result from the uptake of terrestrial material by the forest floor during the tsunami impact. In contrast to T11, the sediment sources near transects T3 and T8 in the harbor area provide fewer sediment sources for the tsunami as the area is built-up or paved (port facilities) resulting in fewer terrestrial input sources and thus a more marine signal.

The Oirase transect represents a further particularity. The profile Oi 5 shows a distinct marine signal of both values, but the other sediment profiles illustrate a low, therefore marine, TAR, but a terrestrial *n*-aldehyde signature.

The natural organic compound approach targeting the differentiation between marine and terrestrial sediment sources illustrates that these indicators can distinguish the tsunami-laid layers from the background sedimentation. However, a differentiation must be made with caution and the context of location-specific parameters in mind. The *n*-alkanes with the calculated TAR indicate a distinct and relatively reliable marine vs. terrestrial source indicator for tsunami deposits across almost all samples. Nonetheless, it must be considered that petrogenic influences could bias the TAR. Further on, *n*-aldehydes are rather variable and more connected to local environmental factors. Therefore, the *n*-aldehydes parameter should only be used under clear location-specific circumstances, while the TAR generally can be used as a reliable indicator for tsunami identification and characterization.

### Petrogenic marker

Destruction as caused by the 2011 Tohoku-oki can lead to the emission of petroleum, oil, and varnish (Stogiannidis & Laane, 2015). Therefore, it can be assumed that in particular fossil petrogenic markers, contaminants are enriched in the tsunami deposits, due to the documented destructive force of the 2011 Tohoku-oki tsunami in Misawa, Futakawame and Oirase (e.g., Misawa City Board of Education, 2012; Nakamura et al., 2012; Nandasena et al., 2012; Hazarika et al., 2013). Thus, petrogenic pollution indicating organic geochemical markers and specific compound ratios have been analysed in this study, comprising *n*-alkane ratios (OEP), petroleum biomarkers (hopanes) and polycyclic aromatic hydrocarbons (PAHs). Corresponding results are summarized in Table 1 and discussed in the following.

**Fig. 6:**
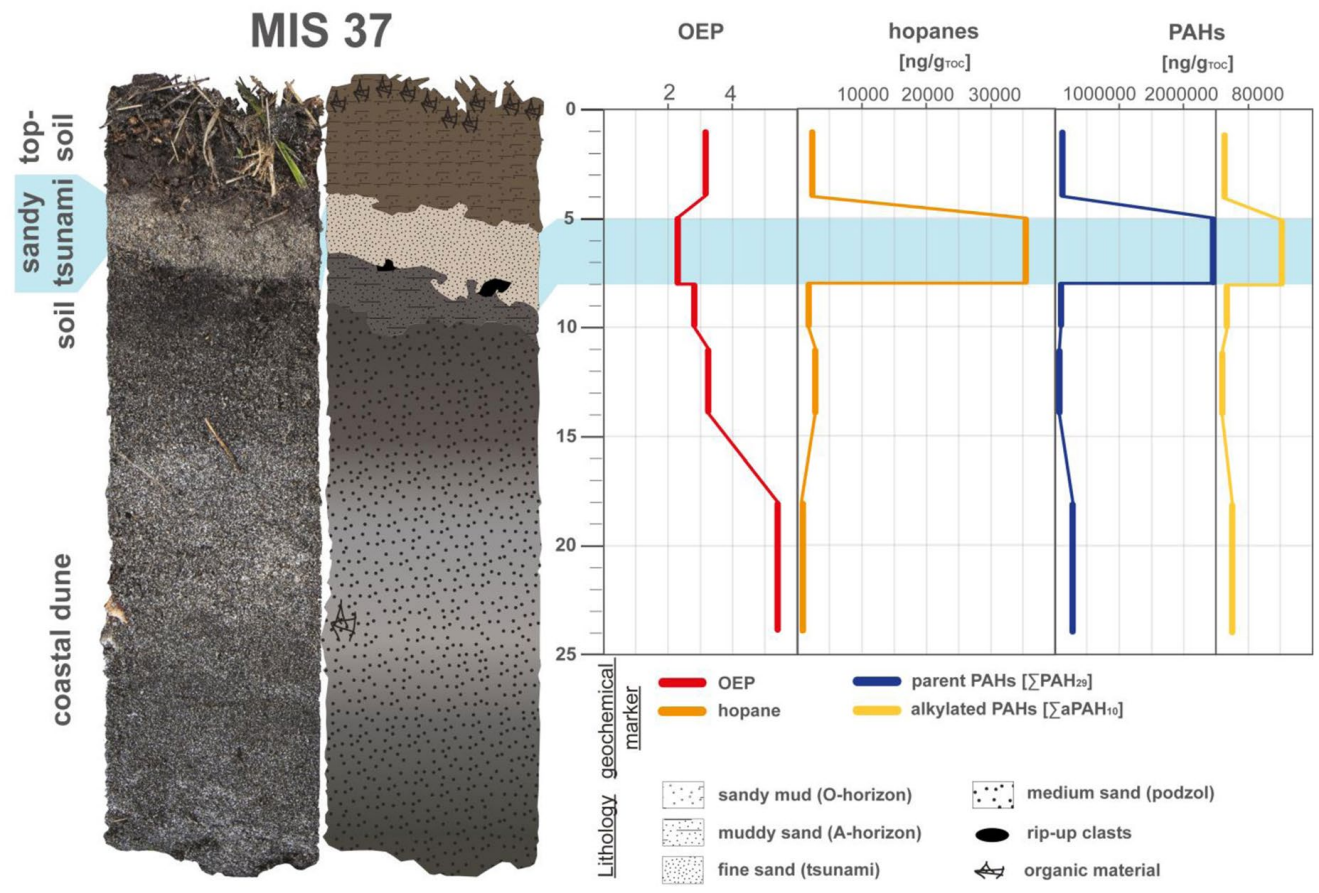
Concentration profiles of the petrogenic compounds; odd-to-even predominance (OEP—*n*-alkanes ratio), hopanes, parent and alkylated polycyclic aromatic hydrocarbons (PAHs) of MIS 37. Data were plotted by using Microsoft Excel 365 and illustrated using Adobe Illustrator

As a first petrogenic indicator, the odd-to-even predominance (OEP) after Peters et al. (2005) reflecting the biogenic or petrogenic source of *n*-alkanes was considered (Eq. 2). Here, a very range of OEP values between 1.3 and 7.3 has been observed (see Table 1). A biogenic origin is indicated by a predominance of odd-numbered *n*-alkanes resulting in higher OEP values, while a petrogenic origin is indicated by mostly even *n*-alkanes and thus lower OEP values (Bellanova et al., 2020; Peters et al., 2005). For the samples from the Aomori coast, the lower OEP values around 2 to 4 indicate a presence of petrogenic pollution in the tsunami samples, while the surrounding deposits present mostly a biogenic signature with higher OEP values. However, few background sediments show shifts to petrogenic signals, which can be related to anthropogenic processes in the harbor or near the Momoishi-Industrial Park (Fig. 6B; Table 1).

2 $$OEP=\frac{{n-C}_{25}+ 6 \times {n-C}_{27}+ {n-C}_{29}}{{4 \times n-C}_{26}+4 \times {n-C}_{28}}\left(n-\mathrm{alkanes}\right)$$

Hopanes have been detected as a second group of petrogenic markers with concentrations of 67 to 15,000 ng/g_TOC_ (see Table 1). These compounds represent fossil organic material as ubiquitous constituents of petroleum and crude oil, and due to their high resistance to biodegradation, they serve as a fingerprint of petrogenic pollution (Silva et al., 2013). However, an increase in the hopanes concentration itself does not directly indicate petroleum contamination, as they can also be formed by biological processes in nature during diagenesis (Sakari, 2012) or microbial processes (Ries-Kautt & Albrecht, 1989). Therefore, maturity indices for hopanes were calculated, for instance 22S/(22S + 22R) C_32_ and 22S/(22S + 22R) C_31_ hopanes (Huang et al., 2014; Peters et al., 2005). Both ratios classify all samples, especially the tsunami ones, to be off fossil nature due to a mature signature (Fig. 8A; Table 1). After oil reached equilibrium, the mature signal does not change and remains constant, indicating an influence of oil and petroleum on the tsunami samples (Peters et al., 2005).

PAHs, both parent and alkylated, are persistent contaminants of fossil fuel-related origin to be found ubiquitously in the studied sediment profiles (Table 2). But noteworthy, PAHs are emitted into the environment not only by petrogenic sources but also by pyrogenic combustion processes. Therefore, PAHs can be divided into these two classes (Stogiannidis & Laane, 2015). Petrogenic PAHs are derived into the environment mainly by petroleum and oils with their unburned derivatives (Ravindra et al., 2008). While pyrogenic PAHs are mainly emitted into the environment as incomplete combustion products of organic matter (coal, wood and fossil fuels; Huang et al., 2014) from stationary sources (e.g., industrial complexes, urban centers and power plants) and mobile sources (e.g., vehicles and ships) (Keshavarzifard et al., 2015). Pyrogenic materials are generally introduced into sediments by atmospheric deposition (Mostert et al., 2010; Schwarzbauer, 2006) and, in the broader context, may be considered ubiquitous background deposition with possible tsunami overprinting. A total of 29 parent (∑PAH_29_) and 10 alkylated PAHs (∑aPAH_10_) were detected in the samples. The ∑PAH_29_ concentration ranges from 1500 to 2,400,000 ng/g_TOC_, while the ∑aPAH_10_ occurs in lower concentrations from 800 to 160,000 ng/g_TOC_ (Table 1). The dominant PAHs in all samples are fluoranthene, pyrene and phenanthrene, as well as the alkylated substitutes methylanthracenes(-phenanthrenes) and methylpyrenes(-fluoranthenes).

**Table 2:** Coordinates of the sediment profiles, distance to the ocean and elevation. Data were plotted by using Microsoft Excel 365

**Transect**	**Sediment profiles**	**Coordinates**	**Distance from coast [m]**	**Elevation [masl]**
**T3**	MIS 37	40° 40.715′ N 141° 25.962′ E	275	8
	MIS 38	40° 40.710′ N 141° 25.943′ E	300	10
	MIS 39	40° 40.711′ N 141° 25.916′ E	325	12
	MIS 40	40° 40.708′ N 141° 25.906′ E	350	13
**T8**	MIS 6	40° 40.987′ N 141° 25.877′ E	275	10
	MIS 15	40° 40.987′ N 141° 25.854′ E	300	12
	MIS 16	40° 40.987′ N 141° 25.836′ E	330.5	13.5
	MIS 17	40° 40.990′ N 141° 25.822′ E	350	15
**T11**	MIS 45	40° 39.500′ N 141° 26.360′ E	230	9
	MIS 46	40° 39.494′ N 141° 26.347′ E	250	9
	MIS 47	40° 39.497′ N 141° 26.328′ E	275	8
**Oi**	Oi 5	40° 37.131′ N 141° 27.355′ E	55	4
	Oi 7	40° 37.111′ N 141° 27.361′ E	60	4
	Oi 8	40° 37.094′ N 141° 27.376′ E	50	4
	Oi 9	40° 37.090′ N 141° 27.365′ E	70	4

**Fig. 7:**
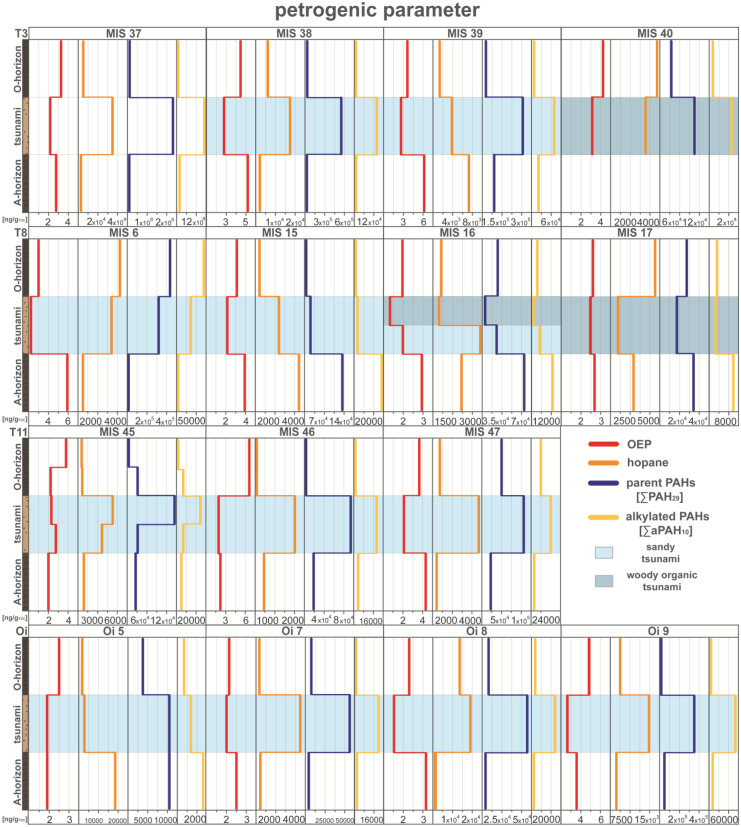
Concentration profiles of the petrogenic compounds; odd-to-even predominance (OEP—*n*-alkanes ratio), hopanes, parent and alkylated polycyclic aromatic hydrocarbons (PAHs) of all sediment profiles ordered by transects. Data were plotted by using Microsoft Excel 365 and illustrated using Adobe Illustrator

The sediment profile MIS 37 (Fig. 6) presents a well-defined petrogenic influence within the tsunami deposits, with both, a low OEP and enrichment in hopanes, PAHs, and alkylated PAHs. This distinct pattern of all proxies suggests a tsunami-driven release of petrogenic substances through the destruction of the harbor area during the tsunami, which was preserved in the sandy tsunami layer (Fig. 6; Bellanova et al., 2021). The distinct signal of petrogenic substances can thus be used to distinguish tsunami and surrounding background layers and aligns with observations of previous studies (e.g., Bellanova et al., 2019, 2020). These observations are also applicable to the other sediment profiles along the studied coastal areas (Fig. 7). This holds true, not only for hopanes, which show a tendency to be increased in almost all tsunami samples, but also for PAHs (both, parent and alkylated), with higher concentrations in almost all tsunami deposits (Fig. 7). An exception to this, however, is transect T8, where the petrogenic indicators tend to be enriched in topsoil (MIS 6) and pre-2011-soil (MIS 15-MIS 17) samples, which might be related to the TOC normalization of these TOC-rich soils.

**Fig. 8:**
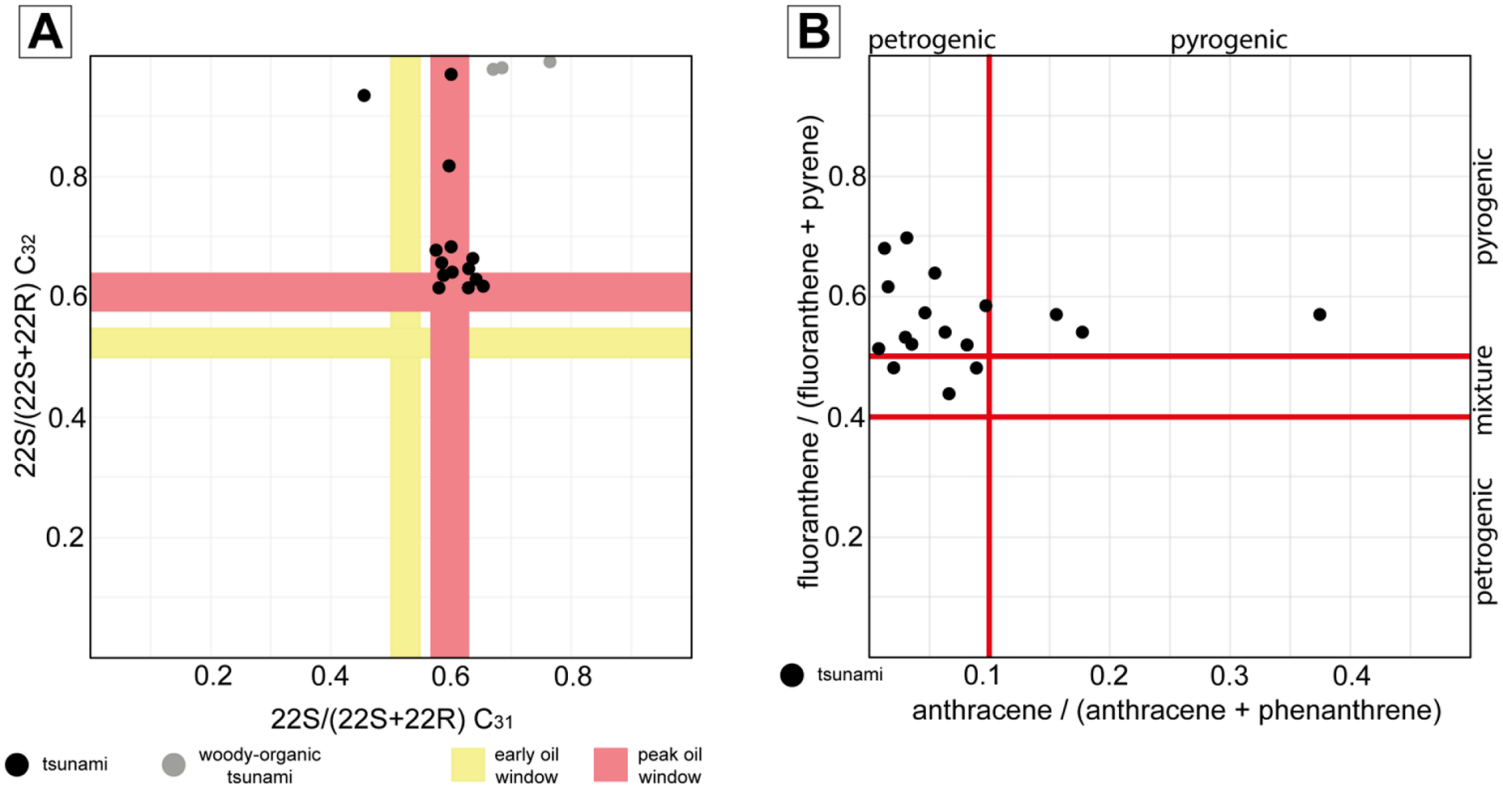
**A** ratios of 22S/(22S + 22R) C_32_ hopanes vs 22S/(22S + 22R) C_31_ hopanes as maturity indicator **B** PAH ratios of F/F + Py vs A/A + P as indicator of petrogenic source. Data were plotted by using Microsoft Excel 365 and illustrated using Adobe Illustrator

The emission sources of the PAHs were evaluated using specific PAH ratios (e.g., Geršlova & Schwarzbauer, 2014; Tobiszewski & Namiesnik, 2012; Yunker et al., 2002) as presented in Table 1, since a distinction between pyrogenic and petrogenic sources solely based on concentrations is difficult (Schwarzbauer et al., 2018). The PAH ratio of anthracene to the sum of anthracene and phenanthrene (A/A + P) covered values between < 0.1 and 0.38, showing a tendency in all samples, particularly in the tsunami deposits, toward a petrogenic origin of the PAHs (Yunker et al., 2002). Only very few outliers, especially in the rural transect T1 (Fig. 8B; Table 1) have been observed. Noteworthy, the particularity in transect T11 with higher ratios (0.18–0.38) can be related to a lack of petroleum emission sources, even during the 2011 tsunami. However, the source determination becomes complex by applying further specific PAH ratios, such as fluoranthene to the sum of fluoranthene and pyrene (F/F + Py). This ratio (Fig. 8B; Table 1) with values from 0.2 to 0.8 indicates a rather pyrogenic pollution source as the origin of the detected compounds. The petrogenic influenced emission by the tsunami shown by the A/A + P ratio is not corroborated by the other indices but is rather complemented by omnipresent background pyrogenic influenced emissions. Therefore, PAH ratios are only semi-quantitative and should primarily be applied when a single emission source dominates the sampling area (Huang et al., 2014).

The considerably higher concentration in the tsunami sediments, in contrast to the surrounding strata, suggests a contamination origin from the destruction by the inundation (Fig. 7). The evidence of an increased input of fossil material is strengthened by the observed enrichment of PAHs. In particular phenanthrene, a petrogenic compound, as does the ratio of A/A + P, represents the introduced fossil material (Yunker et al., 2002). However, a precise source identification for the input of these PAHs in the sampling locations is difficult, because it is very unlikely that there is only a single dominating source.

Overall, petrogenic markers (OEP, hopanes, PAHs) indicate petrogenic contamination released by the destruction of the tsunami landfall and they are subsequently distributed across the affected coastal lowland. This can be related to the observations of destroyed oil tanks and leaking fuel of damaged ships in Misawa harbor (e.g., Misawa City Board of Education, 2012; Bellanova et al., 2021) and the Momoishi-Industrial Park (National Institute for Land and Infrastructure Management, 2011), which could lead to the release of the petrogenic marker into the environment. A certain background pollution in sediments near urban centers, airports (Misawa airport and U.S. air force base) and industry, as present in Misawa, Futakawame and Oirase, is to be expected. Nonetheless, petrogenic indicators allow based on their tsunami-related concentration shifts a distinct identification and characterization of tsunami deposits.

### Sewage-derived marker

During the tsunami landfall, detergent- and sewage-derived compounds can be washed out from flooded sewers or overflowing drainage and irrigation channels and rivers in the proximity of the sample locations (e.g., Mikawame and Futakawame creek). The channels are also potential pathways of road runoff and urban effluents into the Pacific Ocean, which can result from human activity (streets, residential areas and agricultural fields in the hinterland) (Misawa Air Base, 2016). Large-scale flooding and destruction of the infrastructure were reported for all three sampling sites (Misawa City Board of Education, 2012; Nakamura et al., 2012; Nandasena et al., 2012; Hazarika et al., 2013; Tanigawa et al., 2018) during the 2011 Tohoku-oki tsunami. With respect to the irrigation channels, the destruction of floodgates has been documented (Tanigawa et al., 2018). If not compromised, channels and rivers favored the inundation by channeling of the tsunami wave (e.g., at the Misawa River; Koiwa et al., 2014; or the Mikawame creek), which may have led to the introduction and distribution of sewage-derived compounds into the adjacent environment.

Two sewage effluents with recovery potential were detected in the studied sediment profiles: linear alkylbenzenes (LABs) and diisopropylnaphthalenes (DIPNs). The total LAB concentration in the studied sediment profiles is ranging between 21 and 31,000 ng/g_TOC_ and the DIPN concentration ranges 1–3800 ng/g_TOC_ (Table 1). LABs are components in synthetic detergents and are mainly introduced into the environment via municipal sewage and wastewater (Schwarzbauer, 2006). Diisopropylnaphthalenes are technical additives, which find use in coating agents and adhesives (Schwarzbauer, 2006). Furthermore, the substance group is used as a substitute for the banned polychlorinated biphenyls (PCBs). DIPNs are also used in food packaging material (Zhang et al., 2008) and in larger quantities as a dye solvent in a carbonless paper, especially in Japan (Peterman & Delfino, 1990).

**Fig. 9:**
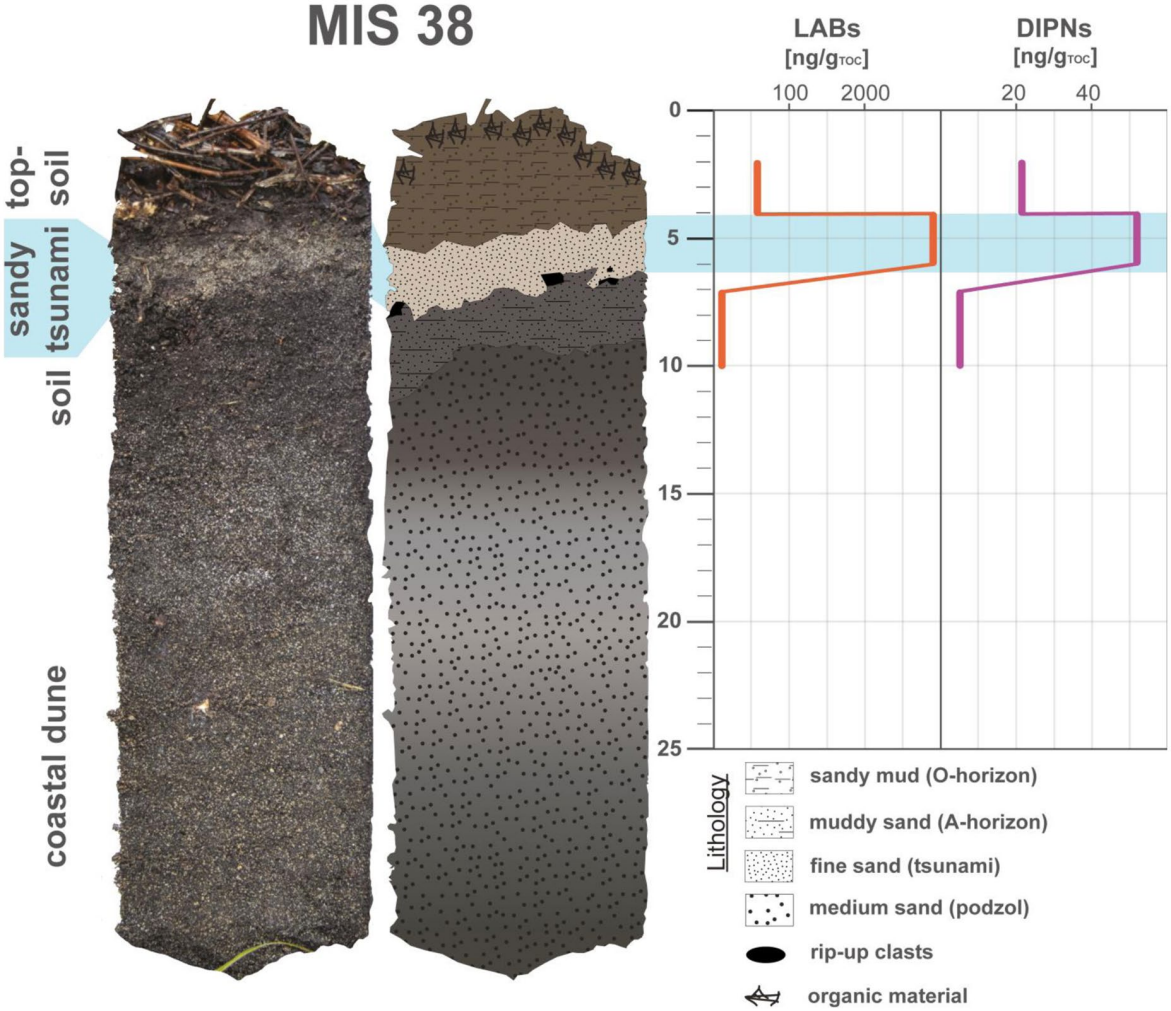
Concentration profiles of sewage-related compounds; linear alkylbenzene (LABs) and diisopropylnaphthalenes (DIPN) of MIS 38. Data were plotted by using Microsoft Excel 365 and illustrated using Adobe Illustrator

**Fig. 10:**
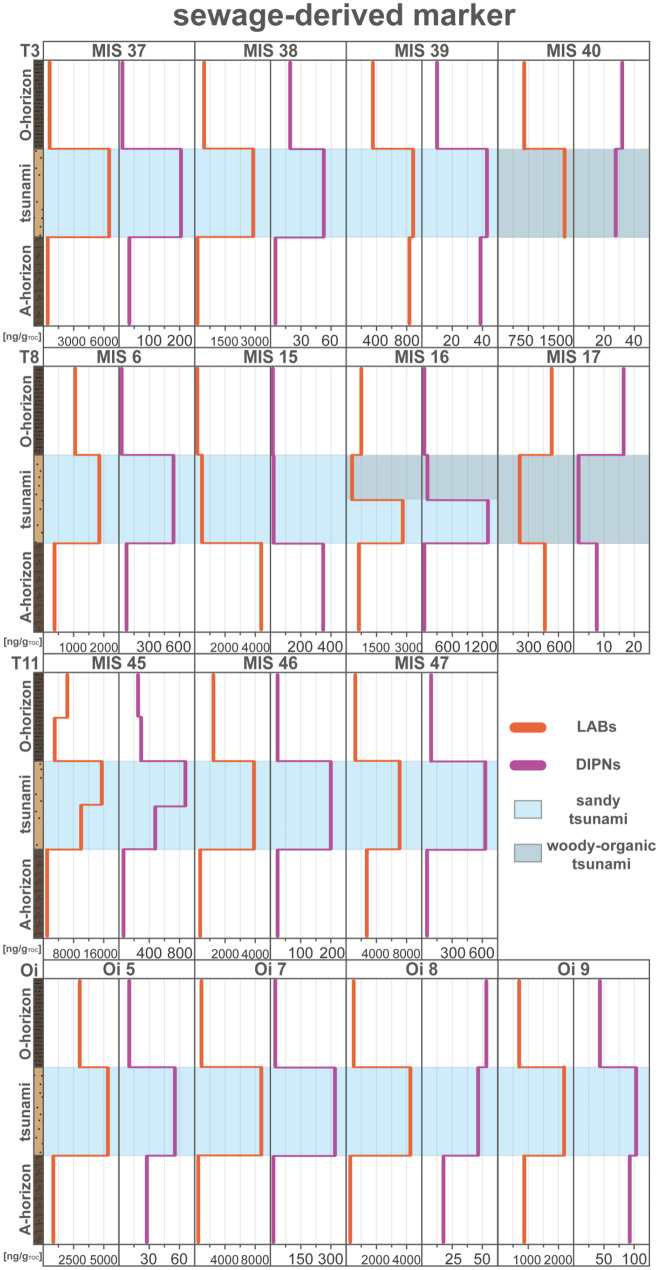
Concentration profiles of sewage-related compounds; linear alkylbenzenes (LABs) and diisopropylnaphthalenes (DIPN) of all sediment profiles ordered by transects. Data were plotted by using Microsoft Excel 365 and illustrated using Adobe Illustrator

The tsunami layer of sediment profile MIS 38 (T3; Fig. 9) in the harbor area shows a distinct enrichment of both sewage-derived contaminants (LABs and DIPNs) in contrast to the surrounding soils. With only few exceptions this general pattern of enriched sewage indicators within the tsunami deposit has been detected in all sediment profiles (Fig. 10). Thereby the LAB (270–16,000 ng/g_TOC_) and DIPN concentration (12–1300 ng/g_TOC_) in the tsunami layers are limited exclusively to the sandy tsunami. The organic-rich woody tsunami deposit presents no distinct concentration variations, which may be related to hydrodynamic processes or the sediment association and absorbability of these compounds.

The high concentrations of sewage-related compounds in the sediment profile of transects T11 and the Oirase transect may be related to their proximity to two drainage and irrigation channels (T11) and near a sewer (Oi), respectively. Noteworthy is also the proximity of the Oirase transect to a food packaging facility in the Momoishi Industrial Park, which may be considered as a potential source when considered to be inundated and flooded by the 2011 Tohoku-oki tsunami. The results further indicate an either periodically release of sewage-related compounds into the environment (maybe by storms or heavy rain events), or a constant but low release via street run-off, as the background soils contain lower but a present background concentration of these sewage parameters. Detergent- and sewage-derived compounds have demonstrated their suitability to identify tsunami deposits from the background sedimentation and allow an evaluation of the destruction’s extent by the inundation of the coastal lowland.

### Old burden marker

Chlorinated substances, such as dichlorodiphenyltrichloroethane (DDT) and associated metabolites (DDD and DDE), referred to as DDX, and polychlorinated biphenyl (PCB), both classified as typical old burden markers (e.g., Kucher et al., 2018; Scheringer, 1999; Schwarzbauer, 2006), were identified in almost all sediment profiles. Concentrations ranged in sum from < LOQ to 1800 ng/g_TOC_ for DDX and from < LOQ to 7500 ng/g_TOC_ for PCBs (see Table 1).

Inundating tsunami waves do not only transport newly through destruction released pollutants, but remobilize persistent organic pollutants (POPs) also by erosion of polluted sediments. Following these old burden substances are remobilized, accumulated, transported and redistributed in the tsunami-laid sediments (e.g., Bellanova et al., 2020). However, it can be assumed, that only low or rather no concentration of old burdens substances, such as DDT and its derivates or PCBs, can be detected in the uppermost topsoil as these contaminants have been prohibited way before the 2011 Tohoku-oki tsunami sediments have been deposited (Ministry of the Environment Japan, 2005). Nevertheless, as described by Bellanova et al. (2020), there is a possibility of soil erosion and remobilization of such contaminants during tsunami inundation, especially in agricultural and industrial areas (e.g., the Sendai Plain). The concept of the old burden marker is based on the hypothesized accumulation within the tsunami sediments, through their uptake during the tsunami via erosion and remobilization of previously polluted sediments.

DDX are known as persistent organic pollutants and belong to the group of xenobiotics (Schwarzbauer, 2006). They were widely used as agricultural pesticides and as a component in anti-fouling paints since the 1940s and generally prohibited in the 1970s and 1980s in Japan, due to their toxicity and persistence in the environment (Ministry of the Environment Japan, 2005; Wu et al., 2016). This persistency allows their identification even decades after the ban (Kang et al., 2016), so that is used as sedimentary anthropogenic markers to this day.

The three detected PCB congener groups (penta-, hexa- and heptachlorobiphenyl), formerly constituents of plasticizer in lacquers, coolants, and hydraulic fluids, were also banned in Japan in the 1980s and replaced by substitutes (Ministry of the Environment Japan, 2005; Schwarzbauer, 2006). Old transformers and other products containing PCB are even today still in use, thus must be considered potential emission sources (Fu et al., 2003; Scheringer, 1999). Unlike DDX, PCBs are ubiquitous contaminants with a wide field of usage and rather not applicable as a specific anthropogenic marker (Lang, 1992; Schwarzbauer, 2006).

**Fig. 11:**
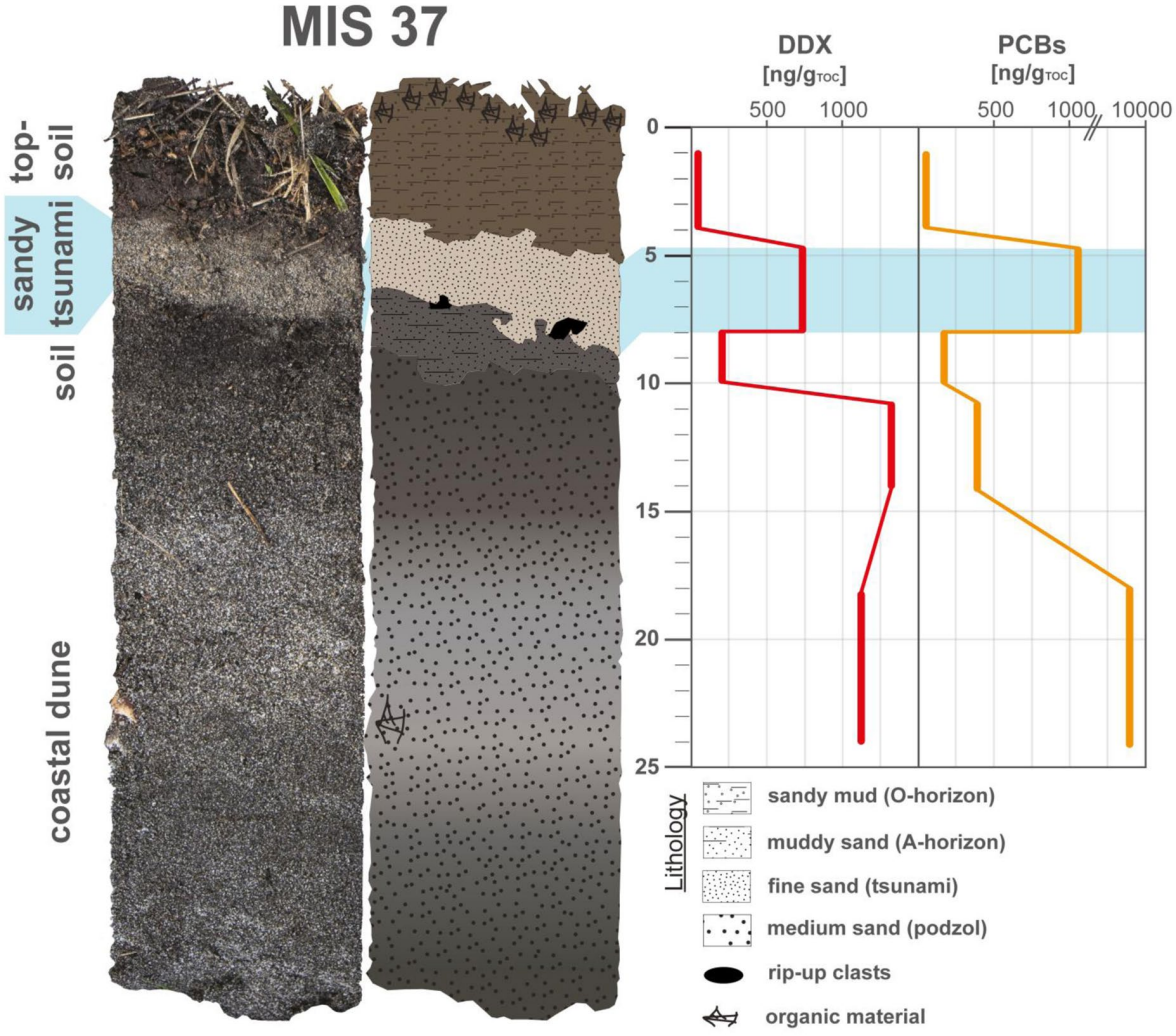
Concentration profiles of chlorinated compounds (old burden); DDX and polychlorinated biphenyls (PCBs) of MIS 37. Data were plotted by using Microsoft Excel 365 and illustrated using Adobe Illustrator

The old burden marker approach for tsunami identification can be illustrated on sediment profile MIS 37, which shows a significant enrichment of both compound groups in the sandy tsunami layer (Fig. 11). However, MIS 37 also shows a potential source of these compounds with an old burden contamination by DDX and PCBs in the paleodune sediments, which could be like to be eroded at locations in the vicinity to the shore where the erosive power of the inundating waves is strongest.

**Fig. 12:**
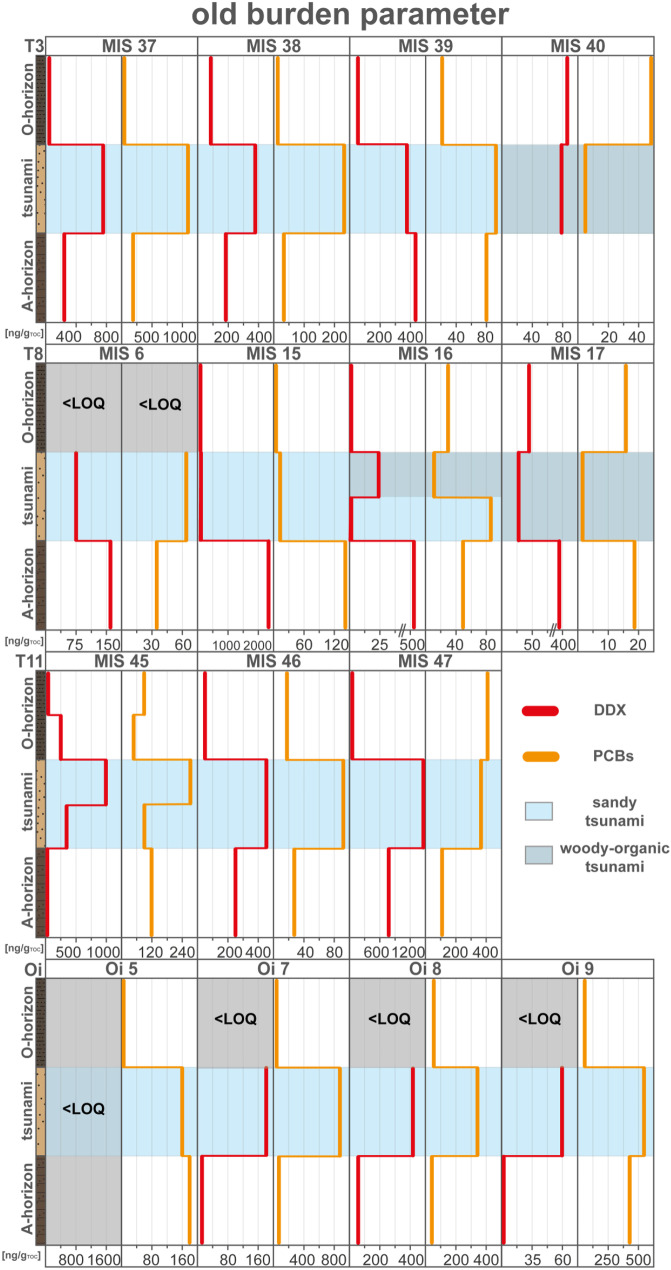
Concentration profiles of chlorinated compounds (old burden); DDX and polychlorinated biphenyls (PCBs) of all sediment profiles ordered by transects. Data were plotted by using Microsoft Excel 365 and illustrated using Adobe Illustrator

The sedimentary inventories of DDX in the sediment profiles range from below the limit of quantification to 2300 ng/g_TOC_ (Table 1). A specific concentration pattern can be traced for most transects with an accumulation of the old burden components mainly in the tsunami deposits closest to the coast (Fig. 12). This may be related to DDX’s sediment association to eroded previously polluted paleodune sediments, which tend to redeposit earlier based on their coarser characteristics (inland fining). Few outliers aside from the tsunami and paleodune deposits show distinct DDX pollution, such as the pre-2011-soil of MIS 15 sediment profile, while the concentration in the topsoils is rather low and concentration peaks can be linked to the TOC normalization. The PCB-congeners are present in concentrations to 7500 ng/g_TOC_ with almost the same concentration distribution pattern as the DDX (Fig. 12; Table 1). Almost all sandy tsunami deposits are significantly enriched in PCBs, and the organic-rich woody tsunami layers are rather depleted in PCBs; this may be also related to the sediment-association of the compounds to the coarser-grained paleodune sediments. For transect T8 is an outlier, with only the tsunami layers of MIS 6 and MIS 16 being enriched.

For most studied transects, it can be concluded that both analyzed POPs are enriched in the sandy tsunami layers. The accumulation of old burdens can therefore be directly related to remobilization and redeposition of the contaminants by the 2011 tsunami as previously described from the Sendai Plain (Bellanova et al., 2020). The most likely sources of these POPs along the Aomori coast can be linked to their former use in the industrial complex, the harbor and in nearby agriculture. This coincides with the observation of significantly enriched POP concentrations in the tsunami deposits of Oirase (industry) and Futakawame (agriculture) transects. An accumulation of these chlorinated substances was also detected in the artificial backfilling of Oi 8 and Oi 9, which could indicate a former emission source in the region. The POPs are suitable indicators for tsunami inundation, as low or no pollution is present in the tsunami surrounding layers, but sufficient pollution sources (harbor and industry complex) and old burdened sediments (Oirase backfilling and paleodune) were identified. When analyzing these compounds their sediment association in relation to the local erosion sources of previously contaminated sediments and here shown inland concentration declines with thinning and fining inland needs to be taken into consideration.

### Synthesis and implications for organic indicators

At first glance, the obtained results of the different organic geochemical approaches (marine vs. terrestrial, petrogenic, detergent-derived, old burdens) applied to the four transects seem—at least in part—contradictory. The depletion of natural terrestrial compounds (*n*-alkanes and *n*-aldehydes, TAR) in tsunami deposits, suggesting a more coastal or marine source of these deposits, counteract the observation of increased anthropogenic pollution (petrogenic, sewage-derived and old burdens from terrestrial sources) within the same tsunami deposits. However, to resolve these initially contradictory seeming results, a closer look into the characteristics of the local conditions, potential sources of specific compounds, the tsunami processes and compound-associated processes need to be considered. Therefore, the compound groups need to be analyzed individually before stitching a broader picture.

Natural organic substances and their ratios (TAR) present sediment-source indicators that help to identify or rather differentiate between marine-dominated or terrestrial-dominated sources of individual deposits (e.g., Alpar et al., 2012; Bellanova et al., 2020; Mathes-Schmidt et al., 2013; Shinozaki et al., 2015). However, these markers are limited when considering mixtures of both environmental domains, as for instance short-chained *n*-alkanes are not exclusively of marine origin but rather omnipresent in the environment (Bellanova et al., 2021). Also, *n*-aldehydes have shown to be not suitable for tsunami identification or characterization in this setting, which may be related to all sediment profiles to be located within or near the coastal control forest, which provides an omnipresent and likely dominating and superimposing source of *n*-aldehydes. Further, ratios, such as the TAR, can be modified by the secondary influence of petrogenic pollution (OEP), which might lead to misinterpretations when not carefully considered. Tsunami-related processes (i.e., erosion, sediment-associated transport, backwash) and secondary effects (leaching) have been documented to modify tsunami deposits (Bellanova et al., 2021; Shinozaki et al., 2015).

Anthropogenic pollutants (e.g., PAHs, hopanes, LABs) are to a lesser extent sediment-source indicators (except for old burdens, DDX, PCBs), but signal the pollution from anthropogenic (mostly terrestrial) sources. This pollution in turn can allow an evaluation of a tsunami’s destructiveness and to trace specific pollutants back to their (potential) point sources (e.g., packaging facilities in Oirase). While some pollutants (PAHs, hopanes, LABs) are independent of terrestrial material through their release by the destruction of infrastructure, buildings and industries, old burdens are limited to erosion and remobilization of previously polluted sediments. Furthermore, Bellanova et al. (2020) noted that these organic compounds are transported in various forms—adsorbed on mineral grains or organic matter, or as free substances in water. This needs to be considered for each individual compound group, as pollution-derived compounds seemed to be rather sediment-associated (especially old burdens) and tend to decrease the further inland the sandy tsunami layer reaches, while these compounds are not to be present at all in the solely terrestrial derived woody-organic tsunami layer. The continuing landward mixture of sediments and thus increasing intake of the terrestrial organic matter reported for tsunami deposits (e.g., Bellanova et al., 2020, 2021; Szczuciński et al., 2012) can also lead to a dilution of specific compounds with a limited point source.

Consequently, the results from the Aomori coast are not contradictive but derive from a complex interplay of compound-specific processes with the inundating tsunami waves that need to be evaluated individually. Overall, depletion of natural terrestrial material can be observed in the tsunami layers, while singularly introduced contaminants from terrestrial anthropogenic sources tend to be enrichment within the same tsunami deposits. By considering the processes affecting a coastal setting during a tsunami (i.e., erosion, mixing, remobilization, backwash), subsequent effect on the environment and sedimentation, both anthropogenic pollutants and natural compounds have proven to be suitable and useful markers to identify but especially to characterize tsunami layers.

For further application especially to paleo and historical tsunamis, the release and usage of compounds is a crucially important factor. Some of the compounds have only been used since industrialization and can only be used for modern tsunamis and thus not for paleo-tsunamis. For paleo-tsunamis and historical tsunamis, the natural substances are a better indicator for identifying and characterizing tsunamis, with the need to add more compounds to get a broader picture of deposited substances.

## Conclusions

In this study, a broad set of natural organic and anthropogenic indicators (e.g., *n*-alkanes, PAHs, hopanes, DDX) have been applied to identify and distinguish tsunami deposits in sedimentary archives from a coastal stretch of ca. 10 km along the Aomori coast affected by the 2011 Tohoku-oki tsunami. Concentration profiles and specific ratios (e.g., TAR, OEP) have been interpreted using two different approaches. Firstly, differentiation of marine and terrestrial matter allowed to characterize the mixing processes of both organic matter fractions due to the tsunami impact. Secondly, linking of constituents to emission sources comprising petrogenic, detergent and sewage-derived pollution as well as old burden pointed not only to a pollution revealed by the tsunami damages but also to dispersion processes, in particular erosion, transport, mixing and redeposition of particle-associated contaminants. Both approaches have been proved to be suitable not only to identify unambiguously tsunamites in sedimentary archives but also to reveal detailed insights into the tsunami-driven dispersion of particle-associated organic matter.

Generally, the organic geochemical proxies as tested in this study can be reliably used to identify and characterize tsunami deposits in the sedimentary record. Further, the results support prior studies on organic geochemical indicators and expand the approach with additional indicative compound groups. And finally, this strategy can be transferred to other locations affected by tsunamis for an in-depth characterization of the destruction and environmental changes induced by tsunami events.

## Supplementary Information

Below is the link to the electronic supplementary material.

Supplementary file1 (DOCX 51994 KB)

## Data Availability

All data that have contributed to the reported results are available from the corresponding author at request.

## References

[CR1] Aloupi M, Angelidis MO (2001). Geochemistry of natural and anthropogenic metals in the coastal sediments of the island of Lesvos, Aegean Sea. Environmental Pollution.

[CR2] Alpar, B., Ünlü, S., Altınok, Y., Özer, N., & Aksu, A. (2012). New approaches in assessment of tsunami deposits in Dalaman (SW Turkey). *Natural Hazard,**60*(1), 27–41. 10.1007/s11069-010-9692-5

[CR3] Arai K, Inoue T, Ikehara K, Sasaki T (2014). Episodic subsidence and active deformation of the forearc slope along the Japan Trench near the epicenter of the 2011 Tohoku Earthquake. Earth and Planetary Science Letters.

[CR4] Bellanova P, Frenken M, Richmond B, Schwarzbauer J, La Selle S-P, Griswold F, Jaffe B, Nelson A, Reicherter K (2019). Organic geochemical investigation of far-field tsunami deposits of the Kahana Valley, O‘ahu, Hawai‘i. Sedimentology.

[CR5] Bellanova P, Frenken M, Reicherter K, Jaffe B, Szczuciński W, Schwarzbauer J (2020). Anthropogenic pollutants and biomarkers for the identification of 2011 Tohoku-oki tsunami deposits (Japan). Marine Geology.

[CR6] Bellanova P, Frenken M, Nishimura Y, Schwarzbauer J, Reicherter K (2021). Tracing woody-organic tsunami deposits of the 2011 Tohoku-oki event in Misawa (Japan). Scientific Reports.

[CR7] Blott SJ, Pye K (2001). GRADISTAT: A grain size distribution and statistics package for the analysis of unconsolidated sediments. Earth Surface Processes Landforms.

[CR8] Blume, H.-P., Brümmer, G.W., Horn, R., Kandeler, E., Kögel-Knabner, I., Kretzschmar, R., Stahr, K., & Wilke, B.-M. (2010). In *Lehrbuch der Bodenkunde* (16th ed., pp. 285). Spektrum Akademischer Verlag Heidelberg. (in German).

[CR9] Bourbonniere RA, Meyers PA (1996). Sedimentary geolipid records of historical changes in the watersheds and productivities of Lakes Ontario and Erie. Limnology and Oceanography.

[CR10] Chagué-Goff C, Andrew A, Szczuciński W, Goff J, Nishimura Y (2012). Geochemical signatures up to the maximum inundation of the 2011 Tohoku-oki tsunami — Implications for the 869 AD Jogan and other palaeotsunamis. Sedimentary Geology.

[CR11] Chagué-Goff C, Szczuciński W, Shinozaki T (2017). Applications of geochemistry in tsunami research: A review. Earth Science Reviews.

[CR12] Chen W-L, Xie Z, Wolschke H, Gandrass J, Kötke D, Winkelmann M, Ebinghaus R (2016). Quantitative determination of ultra-trace carbazoles in sediments in the coastal environment. Chemosphere.

[CR13] Commendatore MG, Nievas ML, Amin O, Esteves JL (2012). Sources and distribution of aliphatic and polyaromatic hydrocarbons in coastal sediments from the Ushuaia Bay (Tierra del Fuego, Patagonia, Argentina). Marine Environment Research.

[CR14] Depietri Y (2020). The social–ecological dimension of vulnerability and risk to natural hazards. Sustainability Science.

[CR15] De Vos B, Vandecasteele B, Deckers J, Muys B (2005). Capability of loss-on-ignition as a predictor of total organic carbon in non-Calcareous forest soils. Communications in Soil Science and Plant Analysis.

[CR16] Dsikowitzky L, Schwarzbauer J, Kronimus A, Littke R (2004). The anthropogenic contribution to the organic load of the Lippe River (Germany). Part 2: Quantification of specific organic contaminants. Chemosphere.

[CR17] Dung TTT, Cappuyns V, Swennen R, Phung NK (2013). From geochemical background determination to pollution assessment of heavy metals in sediments and soils. Reviews in Environmental Science and Biotechnology.

[CR18] Eglinton G, Hamilton RJ, Swan T (1963). The distributions of alkanes. Chemical plant taxonomy.

[CR19] Folk RL, Ward WC (1957). Brazos River bar [Texas]; a study in the significance of grain size parameters. Journal of Sedimentary Research.

[CR20] Fu J, Mai B, Sheng G, Zhang G, Wang X, Peng P, Xiao X, Ran R, Cheng F, Peng X, Wang Z, Wa Tang U (2003). Persistent organic pollutants in environment of the Pearl River Delta, China: An overview. Chemosphere.

[CR21] Gee, G. W., & Or, D. (2002). Particle-size analysis. In J. H. Dane, & G. C. Topp, (Eds.), *Methods of Soil Analysis. Part 4 - Physical methods* (pp. 255–293). Soil Science Society America Book Series 5, Madison, Wisconsin.

[CR22] Geršlova E, Schwarzbauer J (2014). Hydrocarbon based indicators for characterizing potential sources of coal derived pollution in the vicinity of the Ostrava city. Environmental Earth Science.

[CR23] Goodwin, T. W., & Mercer, E. I. (1983). *Introduction to Plant Biochemistry*. (2nd ed., p. 677). Pergamon Press.

[CR24] Goto T, Satake K, Sugai T, Ishibe T, Harada T, Gusman AR (2019). Tsunami history over the past 2000 years on the Sanriku coast, Japan, determined using gravel deposits to estimate tsunami inundation behavior. Sedimentary Geology.

[CR25] Hazarika H, Kasama K, Suetsugu D, Kataoka S, Yasufuku N (2013). Damage to geotechnical structures in waterfront areas of northern Tohoku due to the March 11, 2011 tsunami disaster. Indian Geotechnical Journal.

[CR26] Hinga, B. D. R. (2015). In *Ring of Fire: An Encyclopedia of the Pacific Rim’s Earthquake, Tsunamis, and Volcanoes *(p. 402). ABC-CLIO.

[CR27] Hinrichsen, D. (1999). The coastal population explosion*. *In B. Cicin-Sain, R. W. Knecht, & N. Foster (Eds.),* Trends and Future Challenges for U.S. National Ocean and Coastal Policy *(pp. 27–29). Proceedings of a Workshop, Washington DC, January 22, 1999, (NOAA).

[CR28] Huang L, Chernyak SM, Batterman SA (2014). PAHs (Polycyclic Aromatic Hydrocarbons), Nitro-PAHs, Hopanes and Steranes Biomarkers in Sediments of Southern Lake Michigan, USA. Science of the Total Environment.

[CR29] Huppert HE, Sparks RSJ (2006). Extreme natural hazards: Population growth, globalization and environmental change. Philosophical Transactions of the Royal Society A.

[CR30] International Research Institute of Disaster Science (2013). HFA IRIDeS Review Preliminary Report: Focusing on 2011 Great East Japan Earthquake.

[CR31] Jeng W-L (2007). Aliphatic hydrocarbon concentrations in short sediment cores from the southern Okinawa Trough: Implications for lipid deposition in a complex environment. Continental Shelf Research.

[CR32] Kang L, He Q, He W, Kong X, Liu W, Wu W, Li Y, Lan X, Xu F (2016). Current status and historical variations of DDT-related contaminants in the sediments of Lake Chaohu in China and Their Influencing Factors. Environmental Pollution.

[CR33] Keshavarzifard M, Zakaria MP, Hwai TS, Yusuff FM, Mustafa S (2015). Distributions and source apportionment of sediment-associated polycyclic aromatic hydrocarbons (PAHs) and hopanes in rivers and estuaries of Peninsular Malaysia. Environmental Science and Pollution Research.

[CR34] Koiwa N, Kasai M, Kataoka S, Isono T (2014). Examination of relation with tsunami behavior reconstructed from on-site sequence photographs, topography, and sedimentary deposits from the 2011 Tohoku-oki tsunami on the Kamikita Plain, Japan. Marine Geology.

[CR35] Kucher S, Dsikowitzky L, Ricking M, Sujatha CH, Schwarzbauer J (2018). Degree of phenyl chlorination of DDT-related compounds as potential molecular indicator for industrial DDT emissions. Journal of Hazardous Materials.

[CR36] Lang V (1992). Polychlorinated biphenyls in the environment. Journal of Chromatography.

[CR37] Lilly, A., Baggaley, N., & Yeluripati, J. (2020). *A review of the effects of land use and land management changes on soil organic carbon stocks in hill farms*. Climate-positive farming reviews.

[CR38] Loring DH, Rantala RTT (1992). Manual for the geochemical analyses of marine sediments and suspended particulate matter. Earth-Science Reviews.

[CR39] Mathes-Schmidt M, Schwarzbauer J, Papanikolaou I, Syberberg F, Thiele A, Wittkopp F, Reicherter K (2013). Geochemical and micropaleontological investigations of tsunamigenic layers along the Thracian Coast (Northern Aegean Sea, Greece). Zeitschrift Für Geomorphologie.

[CR40] Meyers PA (2003). Applications of organic geochemistry to paleolimnological reconstructions: A summary of examples from the Laurentian Great Lakes. Organic Geochemistry.

[CR41] Ministry of Environment Japan. (2005). The National Implentation Plan of Japan under Stockholm Comvention on Persistent Organi Pollutants. Inter-Ministerial General Directors’ Meeting on the Stockholm Convention on Persistent Organic Pollutants, 24 June 2005.

[CR42] Misawa Air Base. (2016). *Drinking Water – Consumer Confidence Report (CCR) 2016*. Misawa AB, Japan.

[CR43] Misawa City Board of Education. (2012). *Great East Japan Earthquake Memory of Misawa City*. (in Japanese).

[CR44] Mostert MMR, Ayoko GA, Kokot S (2010). Application of chemometrics to analysis of soil pollutants. Trends in Analytical Chemistry.

[CR45] Müller, S., Wilcke, W., Kanchanakool, N., & Zech, W. (2000). Polycyclic aromatic hydrocarbons (PAHs) and polychlorinated biphenyls (PCBs) in particle-size separates of urban soils in Bangkok, Thailand*. Soil Science,**165*(5), 412–419. 10.1097/00010694-200005000-00005

[CR46] Nakamura Y, Nishimura Y, Putra PS (2012). Local variation of inundation, sedimentary characteristics, and mineral assemblages of the 2011 Tohoku-oki tsunami on the Misawa coast, Aomori, Japan. Sedimentary Geology.

[CR47] Nandasena NAK, Sasaki Y, Tanaka N (2012). Modeling field observations of the 2011 Great East Japan tsunami: Efficacy of artificial and natural structures on tsunami mitigation. Coastal Engineering Journal.

[CR48] National Institute for Land and Infrastructure Management. (2011). Summary of the Field Survey and Research on “The 2011 off the Pacific coast of Tohoku Earthquake” (the Great East Japan Earthquake). *Technical Note of National Institute for Land and Infrastructure Management,**647*.

[CR49] Ostrowska A, Porębska G (2012). Assessment of TOC-SOM and SOM-TOC Conversion in Forest Soil. Polish Journal of Environmental Studies.

[CR50] Peterman, P. H., & Delfino, J. J. (1990). Identification of isopropylbiphenyl, alkyl diphenylmethanes, diisopropylnaphthalene, linear alkyl benzenes and other polychlorinated biphenyl replacement compounds in effluents, sediments and fish in the fox river system, Wisconsin*. Biomedical Environmental Mass Spectrometry,**19*(12), 755–770. 10.1002/bms.1200191203

[CR51] Peters, K. E., Walters, C. C., & Moldowan, J. M. (2005). *The Biomarker Guide: Volume 2, Biomarkers and Isotopes in Petroleum Systems and Earth* (pp. 1132). Cambridge University Press.

[CR52] Prahl, F. G., & Carpenter, R. (1987). Hydrocarbons in Washington coastal sediments. *Estuarine, Coastal and Shelf Sciences,**18*, 703-720.

[CR53] Prahl, F. G., & Pinto, L. A. (1987). A geochemical study of long-chain n-aldehydes in Washington coastal sediments. *Geochimica Cosmochimica Acta,**51*, 1573-1582.

[CR54] Ravindra K, Sokhi R, Van Grieken R (2008). Atmospheric polycyclic aromatic hydrocarbons: Source attribution, emission factors and regulation. Atmospheric Environment.

[CR55] Rieley G, Collier RJ, Jones DM, Eglinton G (1991). The biogeochemistry of Ellesmere Lake, UK I: Source correlation of leaf wax inputs to the sedimentary record. Organic Geochemistry.

[CR56] Ries-Kautt M, Albrecht P (1989). Hopane-derived triterpenoids in soils. Chemical Geology.

[CR57] Roussiez V, Ludwig W, Probst J-L, Monaco A (2005). Background levels of heavy metals in surficial sediments of the Gulf of Lions (NW Mediterranean): An approach based on 133Cs normalization and lead isotope measurements. Environmental Pollution.

[CR58] Sakari, M. (2012). Depositional history of polycyclic aromatic hydrocarbons: reconstruction of petroleum pollution record in Peninsular Malaysia. In T. Puzyn, & A. Mostrag (Eds.), *Organic Pollutants Ten Years After the Stockholm Convention - Environmental and Analytical Update*. IntechOpen.

[CR59] Scheringer M (1999). Persistenz und Reichweite von Umweltchemikalien.

[CR60] Schulte P, Lehmkuhl F (2018). The difference of two laser diffraction patterns as an indicator for post-depositional grain size reduction in loess-paleosol sequences. Palaeogeography, Palaeoclimatology, Palaeoecology.

[CR61] Schwarzbauer J (2006). Organic Contaminants in Riverine and Groundwater Systems - Aspects of the Anthropogenic Contribution.

[CR62] Schwarzbauer J, Littke R, Weigelt V (2000). Identification of specific organic contaminants for estimating the contribution of the Elbe river to the pollution of the German Bight. Organic Geochemistry.

[CR63] Schwarzbauer, J., Stock, F., Brückner, H., Dsikowitzky, L., & Krichel, M. (2018). Molecular organic indicators for human activities in the Roman harbor of Ephesus, Turkey*. Geoarchaeology,**33*(4), 498–509. 10.1002/gea.21669

[CR64] Shiki T, Tachibana T, Fujiwara O, Goto K, Nanayama F, Yamazaki T, Shiki T, Tsuj Y, Minoura K, Yamazaki T (2008). Characteristic Features of Tsunamiites. Tsunamiites—Features and Implications.

[CR65] Shinozaki T, Fujino S, Ikehara M, Sawai Y, Tamura T, Goto K, Sugawara D, Abe T (2015). Marine biomarkers deposited on coastal land by the 2011 Tohoku-oki tsunami. Natural Hazards.

[CR66] Silva TR, Lopes SRP, Spörl G, Knoopers BA, Azevedo DA (2013). Evaluation of anthropogenic inputs of hydrocarbons in sediment cores from a tropical Brazilian estuarine system. Microchemical Journal.

[CR67] Stogiannidis, E., & Laane, R. (2015). Source characterization of polycyclic aromatic hydrocarbons by using their molecular indices: an overview of possibilities. In D. M. Whitacre (Ed.), *Reviews of Environmental Contamination and Toxicology*, *234*, 49–133. 10.1007/978-3-319-10638-0_2 25385513

[CR68] Szczuciński W, Kokociński M, Rzeszewski M, Chagué-Goff C, Cachão M, Goto K, Sugawara D (2012). Sediment sources and sedimentation processes of 2011 Tohoku-oki tsunami deposits on the Sendai Plain, Japan – Insights from diatoms, nannoliths and grain size distribution. Sedimentary Geology.

[CR69] Tanigawa K, Sawai Y, Namegaya Y (2018). Diatom assemblages within tsunami deposit from the 2011 Tohoku-oki earthquake along the Misawa coast, Aomori Prefecture, Northern Japan. Marine Geology.

[CR70] Tipmanee D, Deelaman W, Pongpiachan S, Schwarzer K, Sompongchaiyakul P (2012). Using Polycyclic Aromatic Hydrocarbons (PAHs) as a chemical proxy to indicate Tsunami 2004 backwash in Khao Lak coastal area, Thailand. Natural Hazards and Earth System Science.

[CR71] Tobiszewski M, Namiesnik J (2012). PAH diagnostic ratios for the identification of pollution sources. Environmental Pollution.

[CR72] Tsuji Y, Satake K, Ishibe T, Harada T, Nishiyama A, Kusumoto S (2014). Tsunami heights along the Pacific Coast of Northern Honshu recorded from the 2011 Tohoku and previous great earthquakes. Pure and Applied Geophysics.

[CR73] Van Kaam-Peters HME, Köster J, Van der Gaast SJ, Dekker M, De Leeuw JW, Sinninghe Damste JS (1998). The effect of clay minerals on diasterane/sterane ratios. Geochimica Et Cosmochimica Acta.

[CR74] Wilcke W, Zech W, Kobza J (1996). PAH-Pools in soils along a PAH-deposition gradient. Environmental Pollution.

[CR75] Wilcke W, Amelung W, Zeck W (1997). Heavy metals and polycyclic aromatic hydrocarbons (PAHs) in a rural community leewards of a waste incineration plant. Zeitschrift Für Pflanzenernährung Und Bodenkunde.

[CR76] Wirtz A, Kron W, Löw P, Steuer M (2014). The need for data: Natural disasters and the challenges of database management. Natural Hazards.

[CR77] Wittkopp, F., Reicherter, K., Schwarzbauer, J. (2011). *Organic-geochemical proxys for tsunami events in sediment cores of the Thermaikos Gulf* (pp. 595). Interlaken, Switzerland, 25^th^ International Meeting on Organic Geochemistry (IMOG 2011).

[CR78] Wu C-C, Bao L-J, Tao S, Zeng EY (2016). Significance of antifouling paint flakes to the distribution of dichlorodiphenyltrichloroethanes (DDTs) in estuarine sediment. Environmental Pollution.

[CR79] Yunker M-B, Macdonald R-W, Roxanne V, Mitchell R-H, Goyette D, Sylvestre S (2002). PAHs in the Fraser River basin: A critical appraisal of PAH ratios as indicators of PAH source and composition. Organic Geochemistry.

[CR80] Zhang K, Noonan GO, Begley TH (2008). Determination of 2,6-diisopropylnaphthalene (DIPN) and *n*-dibutylphthalate (DBP) in food and paper packaging materials from US marketplaces. Food Additives and Contaminants Part A.

