# Supplementary material for: Suitable indicators to determine tsunami impact on coastal areas in Northern Japan, Aomori Prefecture
Source: Environ Monit Assess. 2022 Apr 20;194(5):385. doi: 10.1007/s10661-022-09989-4 (PMC9021103; doi:10.1007/s10661-022-09989-4)
Supplement: Supplementary file 1 — Supplementary file1 (DOCX 51994 KB) [file 10661_2022_9989_MOESM1_ESM.docx]

**Supplementary**

**Figure Captions:**

**Fig. S1** Grain size parameter of all samples; bivariate plots of (A) skewness (φ) against sorting (φ); (B) mean (φ) against kurtosis (φ). Data was plotted by using Microsoft Excel 365 and illustrated using Adobe Illustrator.

**Fig.S2** Concentration profiles of the detected natural compounds and ratios, terrigenous/aquatic ratio (TAR - *n*-alkanes ratio) and *n*-aldehydes of all sediment profiles ordered by transects (FGF-normalized). Data was plotted by using Microsoft Excel 365 and illustrated using Adobe Illustrator.

**Fig. S3** Concentration profiles of the petrogenic compounds; odd-to-even predominance (OEP - *n*-alkanes ratio), hopanes, parent and alkylated polycyclic aromatic hydrocarbons (PAHs) of all sediment profiles ordered by transects (FGF-normalized). Data was plotted by using Microsoft Excel 365 and illustrated using Adobe Illustrator.

**Fig. S4** Concentration profiles of sewage-related compounds; linear alkylbenzene (LABs) and diisopropylnaphthalene (DIPN) of all sediment profiles ordered by transects (FGF-normalized). Data was plotted by using Microsoft Excel 365 and illustrated using Adobe Illustrator.

**Fig. S5** Concentration profiles of chlorinated compounds (old burden); DDX and polychlorinated biphenyls (PCBs) of all sediment profiles ordered by transects (FGF-normalized). Data was plotted by using Microsoft Excel 365 and illustrated using Adobe Illustrator.

**Tab. S1**

|  | **concentrations in ng/g_FGF_** | | | | **marine vs. terrestrial approach** | | | **petrogenic parameter** | | | | | | | | **sewage-derived marker** | | **old burden parameter** | |
| --- | --- | --- | --- | --- | --- | --- | --- | --- | --- | --- | --- | --- | --- | --- | --- | --- | --- | --- | --- |
|  | **core** | **description** | **depth [cm]** | **TOC [%]** | ***n*-alkane C_12_-C_32_** | **TAR** | ***n*-aldehyde C_20_-C_32_** | **OEP** | **hopane** | **22S/(22S+22R) C_31_** | **22S/(22S+22R) C_32_** | **PAHs (parent)** | **PAHs (alkylated)** | **A/(A+P)** | **F/(F+Py)** | **LABs** | **DIPNs** | **DDX** | **PCBs** |
| **transect T3** | MIS 37 | topsoil | 1 - 4 | 24.10 | 24000 | 32 | 770 | 3.2 | 140 | 0.6 | 0.6 | 5100 | 570 | 0.04 | 0.6 | 49 | 1.1 | 3.1 | 2.3 |
|  | MIS 37 | tsunami | 5 - 8 | 1.02 | 34000 | 4.3 | 230 | 2.4 | 610 | 0.6 | 0.6 | 42000 | 2800 | 0.01 | 0.7 | 115 | 3.6 | 13 | 20 |
|  | MIS 37 | pre-2011 soil | 8 - 10 | 8.22 | 23000 | 10 | 2200 | 2.7 | 100 | 0.5 | 0.6 | 3500 | 1100 | 0.02 | 0.7 | 33 | 3.1 | 20 | 17 |
|  | MIS 37 | coastal dune | 11 - 14 | 0.23 | 18000 | 29 | 810 | 3.3 | 26 | 0.5 | 0.6 | 320 | 33 | 0.00 | 0.8 | 67 | 3.2 | 15 | 4.5 |
|  | MIS 37 | coastal dune | 18 - 24 | 0.10 | 12000 | 32 | 160 | 5.4 | 0.7 | <LOQ | 1.0 | 2300 | 280 | 0.03 | 0.6 | 757 | 38 | 11 | 75 |
|  | MIS 38 | topsoil | 2 - 4 | 14.48 | 13000 | 11 | 690 | 4.4 | 200 | 0.6 | 0.6 | 1500 | 330 | 0.11 | 0.5 | 24 | 0.9 | 3.4 | 0.5 |
|  | MIS 38 | tsunami | 4 - 6 | 1.50 | 12000 | 4.6 | 290 | 2.8 | 150 | 0.6 | 0.6 | 5100 | 1200 | 0.10 | 0.6 | 27 | 0.5 | 3.6 | 2.2 |
|  | MIS 38 | pre-2011 soil | 7 - 10 | 13.51 | 3400 | 6.2 | 170 | 5.2 | 24 | 0.6 | 0.6 | 2000 | 150 | 0.00 | 0.5 | 6 | 0.4 | 12 | 1.9 |
|  | MIS 38 | coastal dune | 14 - 18 | 0.30 | 2800 | 13 | <LOQ | 4.2 | 8.6 | 0.6 | 0.7 | 460 | 67 | 0.00 | 0.6 | <LOQ | <LOQ | 2.7 | <LOQ |
|  | MIS 39 | topsoil | 0 - 5.5 | 23.46 | 8000 | 4.1 | 1900 | 3.6 | 110 | 0.7 | 1.0 | 1900 | 490 | 0.06 | 0.6 | 29 | 0.8 | 4.2 | 1.7 |
|  | MIS 39 | tsunami | 6 - 8.5 | 5.43 | 10000 | 2.6 | 1400 | 2.6 | 140 | 0.6 | 0.8 | 10000 | 2400 | 0.03 | 0.7 | 29 | 1.5 | 13 | 3.2 |
|  | MIS 39 | pre-2011 soil | 9 - 12 | 6.57 | 28000 | 4.7 | 2200 | 6.2 | 200 | 0.6 | 1.0 | 2600 | 720 | 0.07 | 0.6 | 24 | 1.2 | 13 | 2.4 |
|  | MIS 39 | coastal dune | 15 - 18 | 1.68 | 120000 | 32 | 2800 | 4.8 | 320 | 0.7 | 1.0 | 14000 | 2600 | 0.04 | 0.5 | 87 | 4.6 | 73 | 8.7 |
|  | MIS 40 | topsoil | 0 - 2 | 31.68 | 46000 | 7.6 | 8700 | 4.2 | 760 | 0.8 | 1.0 | 4200 | 340 | 0.92 | 0.0 | 92 | 4.4 | 11 | 8.0 |
|  | MIS 40 | woody-organic tsunami | 2 - 4 | 19.30 | 21000 | 6.5 | 1600 | 3.1 | 280 | 0.8 | 1.0 | 8400 | 1800 | 0.06 | 0.5 | 145 | 2.2 | 6.1 | 0.3 |
|  | MIS 40 | coastal dune top | 6 - 9 | 2.78 | 50000 | 33 | 4100 | 2.9 | 760 | 0.6 | 1.0 | 5500 | 1400 | 0.03 | 0.5 | 56 | 5.9 | 13 | 4.4 |
|  | MIS 40 | coastal dune bottom | 31 - 36 | 0.05 | 4400 | 17 | 430 | 7.3 | 17 | 0.3 | 0.8 | 850 | 150 | 0.04 | 0.6 | 150 | 11 | 1.3 | 0.0 |
| **transect T8** | MIS 6 | topsoil | 0 - 2 | 5.37 | 26000 | 9.4 | 1900 | 3.0 | 130 | 0.7 | 0.9 | 13000 | 2060 | 0.03 | 0.5 | 32 | 0.0 | <LOQ | <LOQ |
|  | MIS 6 | tsunami | 2 - 5 | 1.22 | 2800 | 7.0 | 2200 | 2.2 | 43 | 0.6 | 1.0 | 3700 | 390 | 0.03 | 0.5 | 23 | 6.9 | 0.9 | 0.8 |
|  | MIS 6 | pre-2011 soil | 5 - 9 | 5.43 | 1800 | 10 | 310 | 5.9 | 10 | 0.6 | 0.6 | 150 | 33 | 0.03 | 0.6 | 7.3 | 1.5 | 3.2 | 0.7 |
|  | MIS 6 | coastal dune | 12 - 15 | 0.25 | 64000 | 60 | 22 | 2.9 | 19 | 0.6 | 0.6 | 610 | 86 | 0.02 | 0.6 | 86 | 22 | 2.6 | 1.0 |
|  | MIS 15 | topsoil | 1 - 4 | 17.86 | 5000 | 7.3 | 5 | 3.0 | 39 | 0.6 | 0.7 | 610 | 130 | 0.04 | 0.5 | 8.1 | 0.3 | 1.8 | 0.5 |
|  | MIS 15 | tsunami | 4.5 - 6 | 3.93 | 13000 | 6.4 | 480 | 2.1 | 69 | 0.7 | 0.6 | 640 | 42 | 0.07 | 0.4 | 14 | 0.3 | 0.9 | 0.4 |
|  | MIS 15 | pre-2011 soil | 6 - 9 | 1.78 | 18000 | 14 | 1600 | 3.7 | 41 | 0.7 | 0.8 | 1300 | 290 | 0.02 | 0.6 | 41 | 3.3 | 22 | 1.3 |
|  | MIS 15 | coastal dune | 18 - 20 | 0.10 | 17000 | 23 | 240 | 5.4 | 41 | 0.6 | 0.9 | 2200 | 370 | 0.02 | 0.5 | 150 | 12 | 4.4 | 0.3 |
|  | MIS 16 | topsoil | 0 - 3 | 40.61 | 26000 | 14 | 3000 | 2.0 | 50 | 0.7 | 0.9 | 2900 | 510 | 0.09 | 0.5 | 81 | 2.6 | 0.01 | 3.2 |
|  | MIS 16 | woody-organic tsunami | 2 - 5 | 28.07 | 3200 | 9.1 | 2200 | 1.3 | 34 | 0.7 | 1.0 | 330 | 86 | 0.06 | 0.6 | 24 | 9.5 | 2.1 | 1.2 |
|  | MIS 16 | tsunami | 4 - 6 | 4.94 | 23000 | 9.0 | 2600 | 2.0 | 180 | 0.5 | 0.9 | 1400 | 290 | 0.05 | 0.6 | 150 | 73 | 0.01 | 4.6 |
|  | MIS 16 | pre-2011 soil | 7 - 9 | 2.46 | 4000 | 12 | 1400 | 3.0 | 19 | 0.6 | 0.9 | 640 | 110 | 0.02 | 0.6 | 5.9 | 0.1 | 4.6 | 0.4 |
|  | MIS 17 | topsoil | 0 -2 | 38.27 | 31000 | 11 | 4200 | 2.5 | 350 | 0.8 | 1.0 | 1700 | 240 | 0.03 | 0.6 | 33 | 1.0 | 2.9 | 1.0 |
|  | MIS 17 | woody-organic tsunami | 2 - 5.5 | 37.85 | 8600 | 6.1 | 850 | 2.5 | 73 | 0.7 | 1.0 | 1400 | 310 | 0.02 | 0.6 | 17 | 0.1 | 2.3 | 0.2 |
|  | MIS 17 | pre-2011 soil | 7 - 9 | 11.26 | 14000 | 7.3 | 1100 | 2.7 | 120 | 0.5 | 1.0 | 1400 | 390 | 0.01 | 0.6 | 19 | 0.3 | 16 | 0.8 |
|  | MIS 17 | coastal dune | 12 - 15 | 1.47 | 11000 | 7.0 | 80 | 2.9 | 34 | 0.4 | 1.0 | 3400 | 270 | 0.01 | 0.7 | 58 | 11 | 3.8 | 6.3 |
|  | MIS 17 | coastal dune | 24 - 27 | 0.08 | 4300 | 8.4 | <LOQ | 3.6 | 31 | 0.4 | 0.7 | 460 | 150 | 0.27 | 0.6 | 76 | 0.3 | 0.0 | 1.2 |

**Tab. S1 (continued)**

|  | | **concentrations in ng/g_FGF_** | | | | | | | | **marine vs. terrestrial approach** | | | | | | **petrogenic parameter** | | | | | | | | | | | | | | | | **sewage-derived marker** | | | | **old burden parameter** | | | |
| --- | --- | --- | --- | --- | --- | --- | --- | --- | --- | --- | --- | --- | --- | --- | --- | --- | --- | --- | --- | --- | --- | --- | --- | --- | --- | --- | --- | --- | --- | --- | --- | --- | --- | --- | --- | --- | --- | --- | --- |
|  | | **core** | | **description** | | **depth [cm]** | | **TOC [%]** | | ***n*-alkane C_12_-C_32_** | | **TAR** | | ***n*-aldehyde C_20_-C_32_** | | **OEP** | | **hopane** | | **22S/(22S+22R) C_31_** | | **22S/(22S+22R) C_32_** | | **PAHs (parent)** | | **PAHs (alkylated)** | | **A/(A+P)** | | **F/(F+Py)** | | **LABs** | | **DIPNs** | | **DDX** | | **PCBs** | |
| **transect T11** | | MIS 45 | | topsoil | | 0-1 | | 26.14 | | 15000 | | 2.9 | | 3000 | | 3.8 | | 2.5 | | 0.5 | | 0.5 | | 800 | | 250 | | 0.09 | | 0.6 | | 1700 | | 63 | | 18 | | 26 | |
|  |  | MIS 45 | | topsoil | | 2 - 4.5 | | 0.22 | | 650 | | 6.6 | | 520 | | 2.1 | | 0.7 | | 0.6 | | 0.7 | | 74 | | 17 | | 0.17 | | 0.5 | | 7.7 | | 0.7 | | 0.6 | | 0.1 | |
|  |  | MIS 45 | | tsunami | | 7.5 - 9.5 | | 0.08 | | 15000 | | 10 | | 4400 | | 2.2 | | 33 | | 0.6 | | 0.7 | | 860 | | 170 | | 0.18 | | 0.5 | | 97 | | 5.5 | | 6.3 | | 1.7 | |
|  |  | MIS 45 | | tsunami | | 13 - 18 | | 0.10 | | 33000 | | 27 | | 2000 | | 2.8 | | 33 | | 0.6 | | 0.7 | | 320 | | 65 | | 0.38 | | 0.6 | | 110 | | 5.8 | | 3.2 | | 0.9 | |
|  |  | MIS 45 | | pre-2011 soil | | 21 - 26 | | 5.25 | | 30000 | | 9.5 | | 10000 | | 2.0 | | 48 | | 0.7 | | 0.9 | | 2200 | | 400 | | 0.09 | | 0.7 | | 34 | | 4.5 | | 0.1 | | 9.2 | |
|  |  | MIS 45 | | coastal dune | | 26 - 29.5 | | 0.83 | | 130000 | | 20 | | 32000 | | 1.8 | | 340 | | 0.6 | | 0.9 | | 38000 | | 10000 | | 0.09 | | 0.5 | | 160 | | 17 | | 120 | | 26 | |
|  |  | MIS 46 | | topsoil | | 0 - 4 | | 27.60 | | 140000 | | 23 | | 8000 | | 6.6 | | 19 | | 0.6 | | 0.7 | | 670 | | 170 | | 0.02 | | 0.6 | | 240 | | 4.2 | | 20 | | 3.7 | |
|  |  | MIS 46 | | tsunami | | 7 - 10 | | 0.27 | | 20000 | | 18 | | 4300 | | 2.0 | | 39 | | 0.6 | | 0.7 | | 1700 | | 340 | | 0.04 | | 0.5 | | 71 | | 3.6 | | 8.9 | | 1.6 | |
|  |  | MIS 46 | | pre-2011 soil | | 12 - 16 | | 6.05 | | 10000 | | 13 | | 2900 | | 2.3 | | 24 | | 0.6 | | 0.8 | | 900 | | 180 | | 0.13 | | 0.6 | | 10 | | 1.0 | | 15 | | 2.5 | |
|  |  | MIS 47 | | topsoil | | 0 - 3.5 | | 6.84 | | 8600 | | 1.7 | | 2900 | | 3.7 | | 34 | | 0.6 | | 0.6 | | 2000 | | 520 | | 0.03 | | 0.6 | | 59 | | 3.6 | | 5.1 | | 17 | |
|  |  | MIS 47 | | tsunami | | 3.5 - 5 | | 0.35 | | 30000 | | 11 | | 4900 | | 2.1 | | 67 | | 0.6 | | 0.7 | | 1500 | | 340 | | 0.16 | | 0.6 | | 99 | | 8.9 | | 25 | | 5.2 | |
|  |  | MIS 47 | | pre-2011 soil | | 7 - 11 | | 6.21 | | 110000 | | 17 | | 16000 | | 4.3 | | 31 | | 0.7 | | 0.9 | | 2600 | | 530 | | 0.11 | | 0.7 | | 300 | | 7.0 | | 100 | | 12 | |
| **transect Oi** | | Oi 5 | | topsoil | | 0 - 4 | | 10.87 | | 120000 | | 33 | | 3800 | | 2.5 | | 89 | | 0.6 | | 0.8 | | 330 | | 87 | | 0.05 | | 0.6 | | 290 | | 1.2 | | <LOQ | | 0.2 | |
|  |  | Oi 5 | | tsunami | | 4 - 6 | | 2.03 | | 12000 | | 2.0 | | 750 | | 2.0 | | 88 | | 0.6 | | 0.6 | | 480 | | 55 | | 0.01 | | 0.5 | | 250 | | 2.5 | | <LOQ | | 7.2 | |
|  |  | Oi 5 | | pre-2011 soil | | 13 - 18 | | 1.59 | | 130000 | | 13 | | 1700 | | 2.0 | | 390 | | 0.6 | | 0.7 | | 230 | | 60 | | 0.04 | | 0.6 | | 20 | | 0.6 | | <LOQ | | 3.7 | |
|  |  | Oi 5 | | coastal dune | | 21 - 25 | | 0.39 | | 8900 | | 16 | | 51 | | 1.8 | | 12 | | 0.5 | | 0.6 | | 720 | | 76 | | 0.02 | | 0.6 | | 60 | | 1.3 | | <LOQ | | 1.7 | |
|  |  | Oi 7 | | pre-2011 soil | | 0 -2 | | 15.45 | | 69000 | | 18 | | 4300 | | 1.7 | | 35 | | 0.7 | | 0.8 | | 810 | | 84 | | 0.01 | | 0.6 | | 77 | | 1.6 | | <LOQ | | 4.0 | |
|  |  | Oi 7 | | tsunami | | 4 - 7 | | 0.47 | | 16000 | | 2 | | 1600 | | 1.5 | | 68 | | 0.6 | | 0.6 | | 900 | | 300 | | 0.09 | | 0.5 | | 130 | | 5.1 | | 2.8 | | 13 | |
|  |  | Oi 7 | | pre-2011 soil | | 13 - 17 | | 11.21 | | 14000 | | 21 | | 1300 | | 2.3 | | 29 | | 0.6 | | 0.8 | | 370 | | 98 | | 0.02 | | 0.6 | | 22 | | 1.0 | | 0.6 | | 6.1 | |
|  |  | Oi 8 | | topsoil | | 0 - 4 | | 1.89 | | 200000 | | 22 | | 520 | | 3.5 | | 670 | | 0.6 | | 0.6 | | 330 | | 97 | | 0.07 | | 0.5 | | 23 | | 2.5 | | <LOQ | | 2.3 | |
|  |  | Oi 8 | | tsunami | | 8 - 13 | | 0.29 | | 15000 | | 4.6 | | 2600 | | 1.9 | | 140 | | 0.6 | | 0.6 | | 450 | | 180 | | 0.08 | | 0.5 | | 33 | | 0.4 | | 3.2 | | 2.5 | |
|  |  | Oi 8 | | pre-2011 soil | | 13 - 17 | | 1.58 | | 4000 | | 1.2 | | 1100 | | 5.4 | | 3.1 | | 0.6 | | 0.6 | | 250 | | 79 | | 0.04 | | 0.5 | | 14 | | 0.9 | | 3.4 | | 2.2 | |
|  |  | Oi 8 | | artificial | | 22 - 27 | | 0.39 | | 3100 | | 0.2 | | 330 | | 3.5 | | 2.9 | | 0.6 | | 0.6 | | 2200 | | 660 | | 0.08 | | 0.5 | | 24 | | 1.2 | | 6.6 | | 12 | |
|  |  | Oi 9 | | topsoil | | 0 -2 | | 9.12 | | 140000 | | 14 | | 2600 | | 3.8 | | 350 | | 0.5 | | 0.8 | | 140 | | 78 | | 0.28 | | 0.2 | | 73 | | 3.8 | | <LOQ | | 4.3 | |
|  |  | Oi 9 | | tsunami | | 6 - 8 | | 1.04 | | 41000 | | 5.3 | | 15000 | | 2.2 | | 360 | | 0.6 | | 0.6 | | 8600 | | 1900 | | 0.02 | | 0.5 | | 55 | | 2.5 | | 1.5 | | 13 | |
|  |  | Oi 9 | | pre-2011 soil | | 10 -14 | | 3.34 | | 25000 | | 11 | | 8800 | | 3.3 | | 55 | | 0.6 | | 0.7 | | 1100 | | 180 | | 0.01 | | 0.9 | | 21 | | 2.1 | | 0.1 | | 9.6 | |
|  |  | Oi 9 | | artificial | | 18 -25 | | 0.41 | | 14000 | | 0.5 | | 200 | | 1.7 | | 15 | | 0.6 | | 0.6 | | 4800 | | 710 | | 0.01 | | 0.4 | | 85 | | 0.8 | | <LOQ | | 51 | |

∑PAH_29_: naphthalene; acenaphthylene; biphenyl; acenaphthene; fluorene; dibenzofuran; phenanthrene; anthracene; fluoranthene; pyrene; benzo[ghi]fluoranthene; benzo[c]phenanthrene; benz[a]anthracene; chrysene/triphenylene; cyclopenta[cd]pyrene; benzo[b]fluoranthene; benzo[k]fluoranthene; benzo[j]fluoranthene; benzo[e]pyrene; benzo[a]pyrene; perylene; dibenz[a,j]anthracene; indeno[1,2,3-cd]pyrene; dibenz[a,c]anthracene; dibenz[a,h]anthracene; benzo[b]chrysene; picene; benzo[ghi]perylene; anthanthrene

∑aPAH_10_: methylanthracene(-phenanthrene); dimethylanthracene(-phenanthrene); methylpyrene(-fluoranthene); dimethylpyrene(-fluoranthene); methylbenzanthracen(-phenanthrene); dimethylbenzo(a)anthracen(-phenanthrene); methylbenzo(a)pyrene(-fluoranthene); methylbenzpyrene(-fluoranthene); methylnaphthalene; dimethylnaphthalene

Appendix


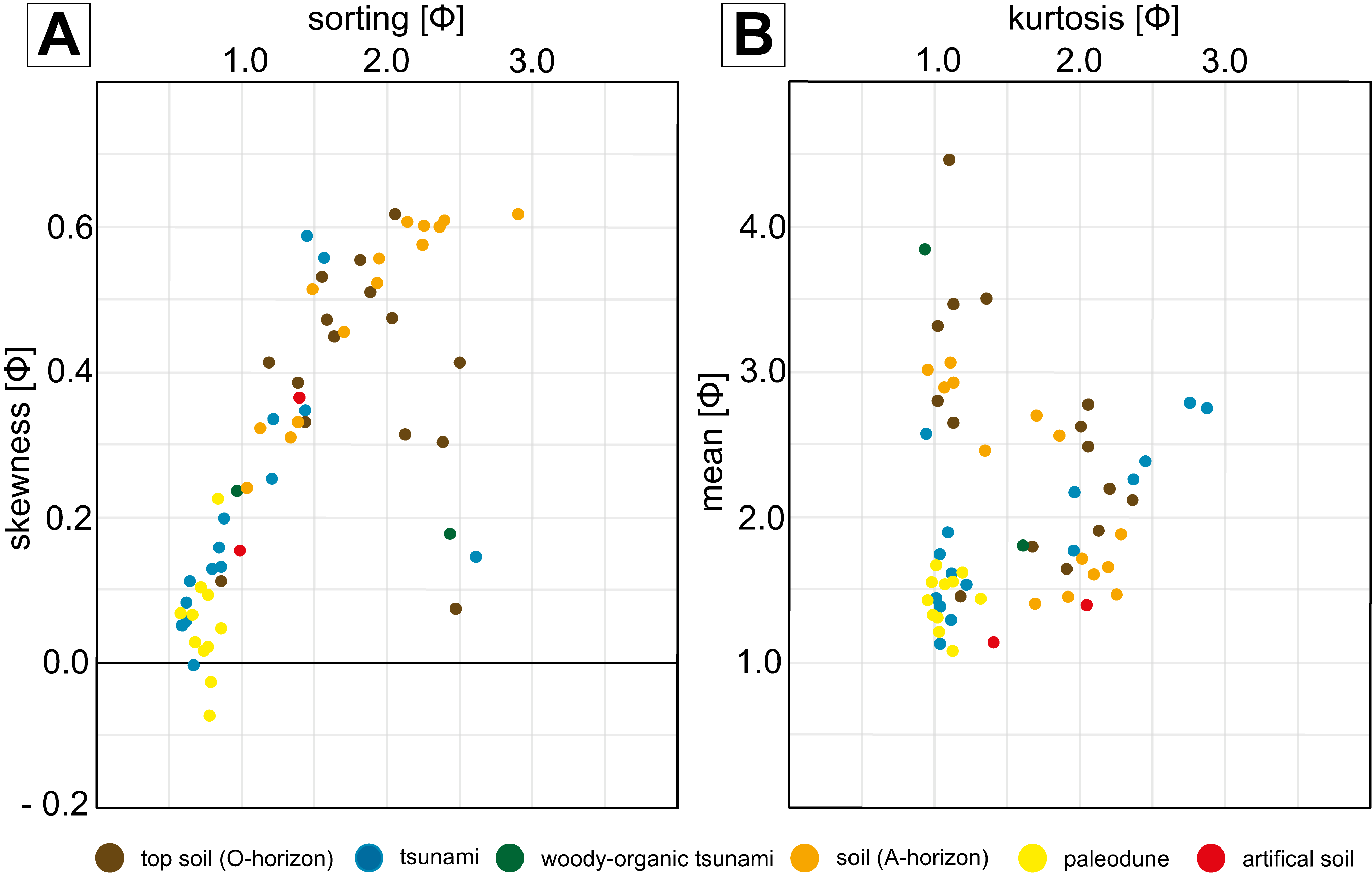


Fig. S1


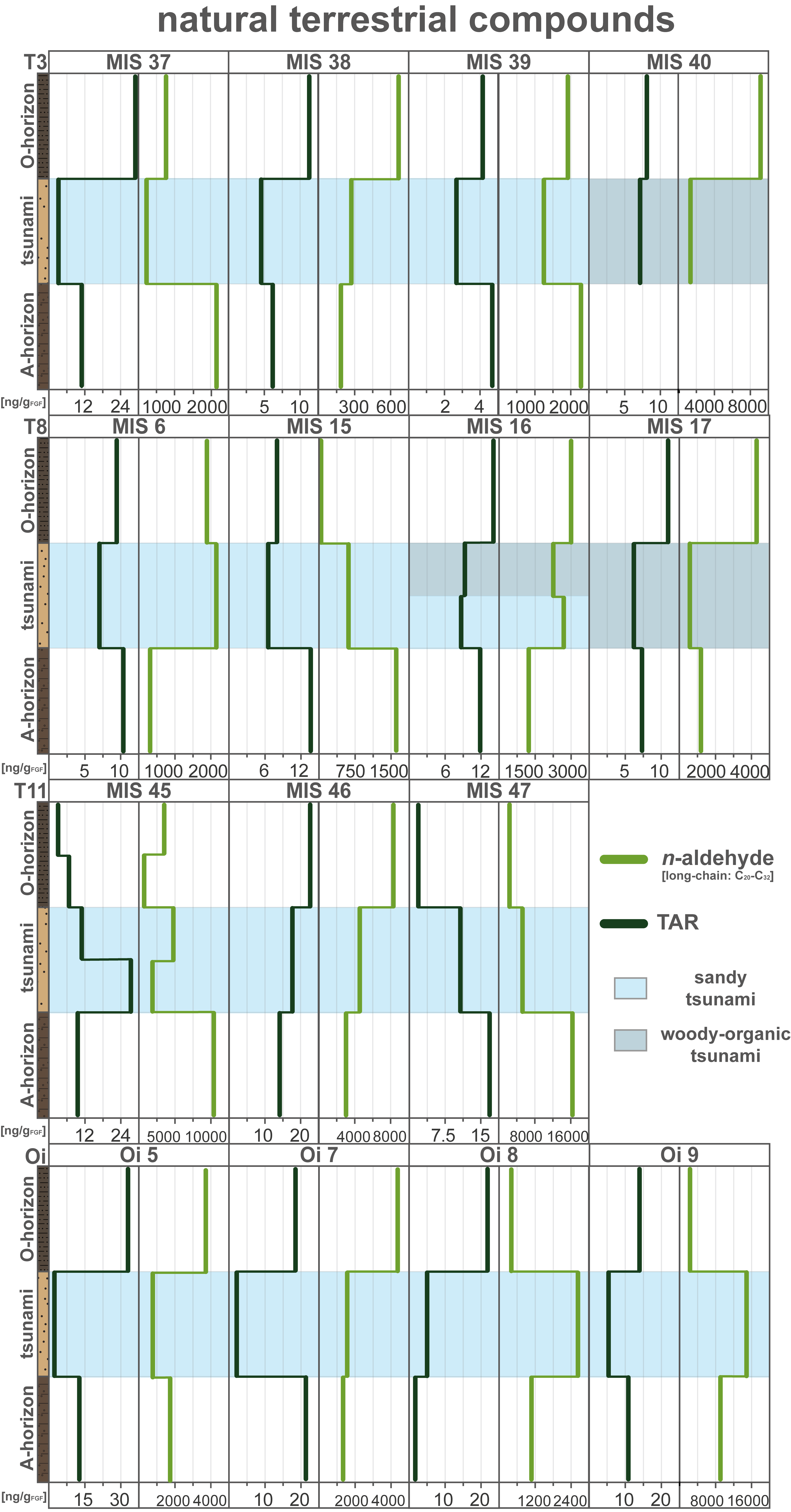
 Fig. S2


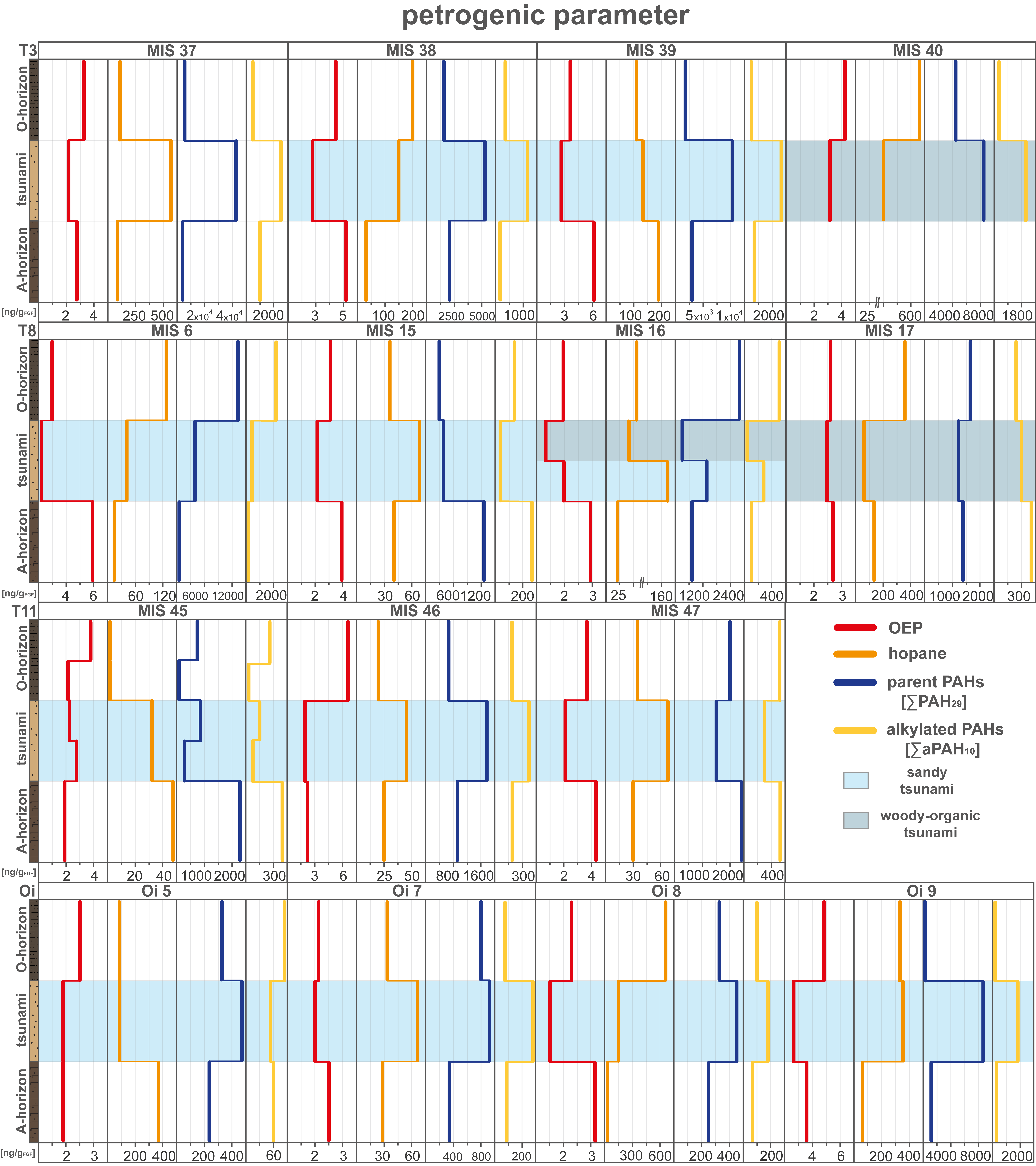


Fig. S3


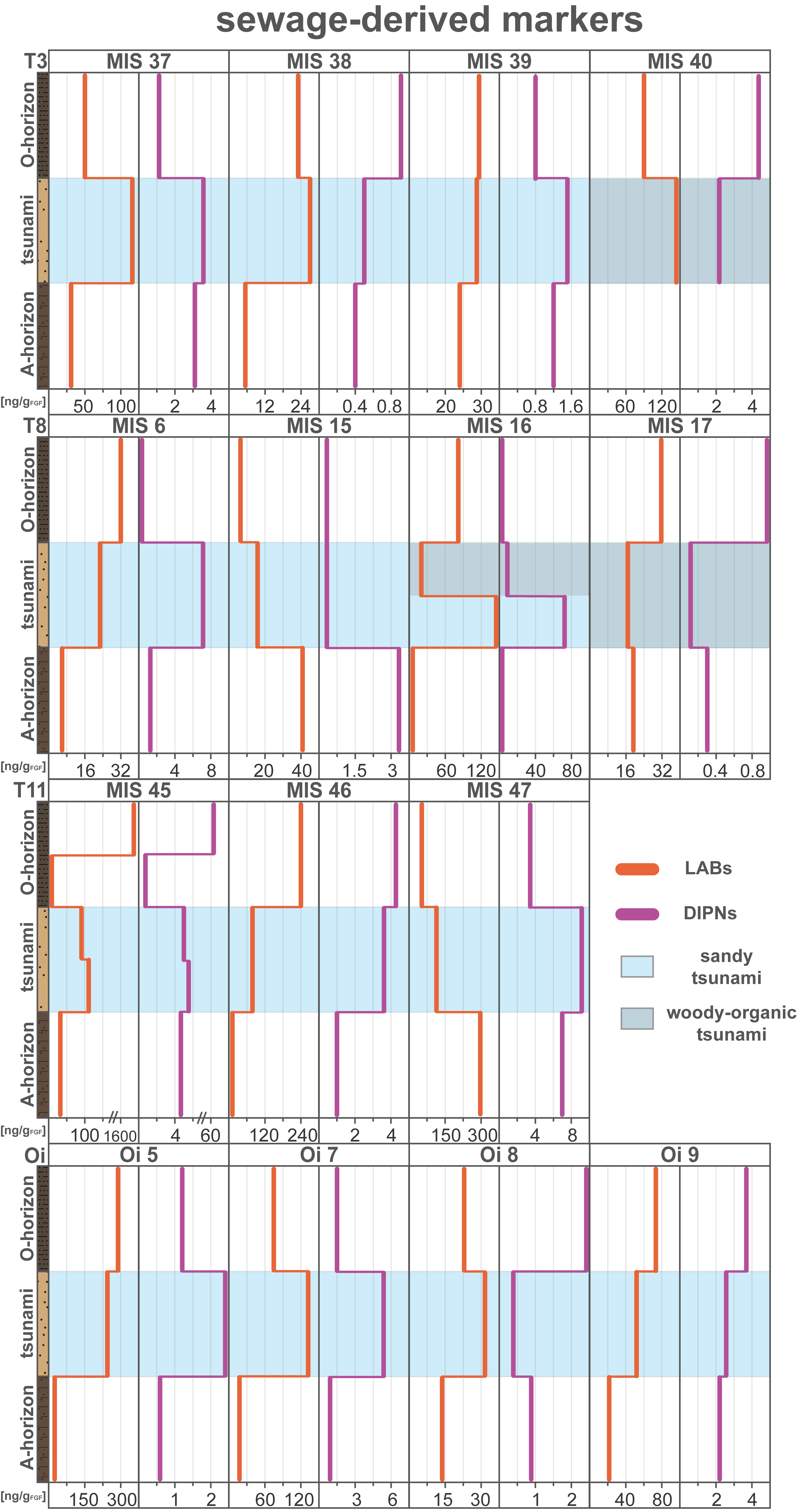
 Fig. S4


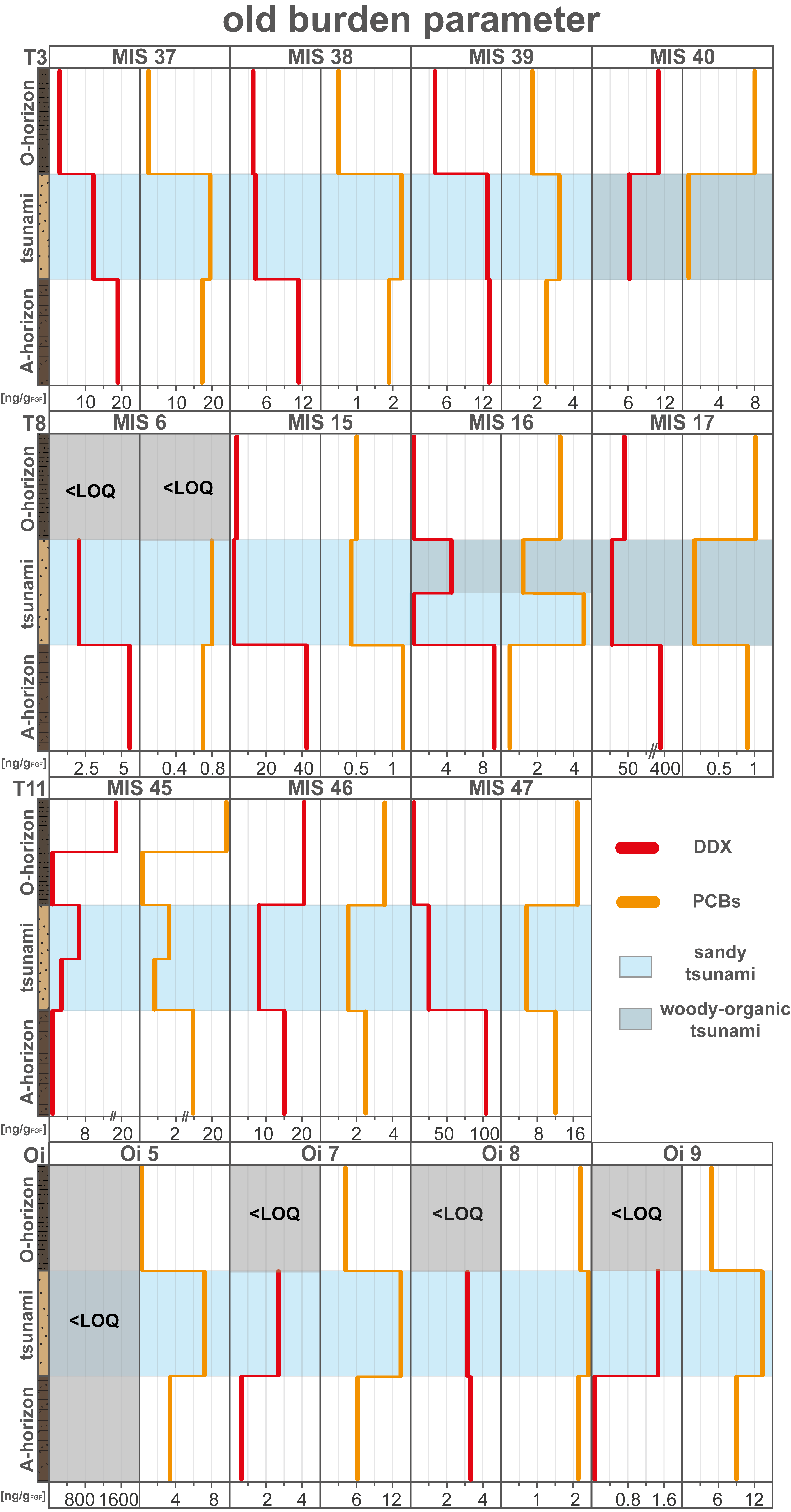
 Fig. S5
